# Agent-Based Model of Combined Community- and Jail-Based Take-Home Naloxone Distribution

**DOI:** 10.1001/jamanetworkopen.2024.48732

**Published:** 2024-12-10

**Authors:** Eric Tatara, Jonathan Ozik, Harold A. Pollack, John A. Schneider, Samuel R. Friedman, Nina T. Harawa, Basmattee Boodram, Elizabeth Salisbury-Afshar, Anna Hotton, Larry Ouellet, Mary Ellen Mackesy-Amiti, Nicholson Collier, Charles M. Macal

**Affiliations:** 1 Decision and Infrastructure Sciences Division, Argonne National Laboratory, Lemont, Illinois; 2 Consortium for Advanced Science and Engineering, The University of Chicago, Chicago, Illinois; 3 Department of Public Health Sciences, The University of Chicago, Chicago, Illinois; 4 Northwestern-Argonne Institute for Science and Engineering, Evanston, Illinois; 5 Crown Family School of Social Work, Policy, and Practice, The University of Chicago, Chicago, Illinois; 6 Urban Health Lab, The University of Chicago, Chicago, Illinois; 7 Department of Medicine, The University of Chicago, Chicago, Illinois; 8 Chicago Center for HIV Elimination, Department of Medicine, The University of Chicago, Chicago, Illinois; 9 Center for Opioid Epidemiology and Policy, Department of Population Health, New York University (NYU) Grossman School of Medicine, New York; 10 Center for Drug Use and HIV Research, NYU School of Global Public Health, New York; 11 Fielding School of Public Health, UCLA (University of California, Los Angeles); 12 David Geffen School of Medicine at UCLA; 13 College of Medicine, Charles R. Drew University of Medicine and Science, Los Angeles, California; 14 Division of Community Health Sciences, School of Public Health, The University of Illinois, Chicago; 15 Department of Family Medicine and Community Health, University of Wisconsin at Madison, Madison; 16 Division of Epidemiology and Biostatistics, School of Public Health, University of Illinois at Chicago, Chicago

## Abstract

**Importance:**

Opioid-related overdose accounts for almost 80 000 deaths annually across the US. People who use drugs leaving jails are at particularly high risk for opioid-related overdose and may benefit from take-home naloxone (THN) distribution.

**Objective:**

To estimate the population impact of THN distribution at jail release to reverse opioid-related overdose among people with opioid use disorders.

**Design, Setting, and Participants:**

This study developed the agent-based Justice-Community Circulation Model (JCCM) to model a synthetic population of individuals with and without a history of opioid use. Epidemiological data from 2014 to 2020 for Cook County, Illinois, were used to identify parameters pertinent to the synthetic population. Twenty-seven experimental scenarios were examined to capture diverse strategies of THN distribution and use. Sensitivity analysis was performed to identify critical mediating and moderating variables associated with population impact and a proxy metric for cost-effectiveness (ie, the direct costs of THN kits distributed per death averted). Data were analyzed between February 2022 and March 2024.

**Intervention:**

Modeled interventions included 3 THN distribution channels: community facilities and practitioners; jail, at release; and social network or peers of persons released from jail.

**Main Outcomes and Measures:**

The primary outcome was the percentage of opioid-related overdose deaths averted with THN in the modeled population relative to a baseline scenario with no intervention.

**Results:**

Take-home naloxone distribution at jail release had the highest median (IQR) percentage of averted deaths at 11.70% (6.57%-15.75%). The probability of bystander presence at an opioid overdose showed the greatest proportional contribution (27.15%) to the variance in deaths averted in persons released from jail. The estimated costs of distributed THN kits were less than $15 000 per averted death in all 27 scenarios.

**Conclusions and Relevance:**

This study found that THN distribution at jail release is an economical and feasible approach to substantially reducing opioid-related overdose mortality. Training and preparation of proficient and willing bystanders are central factors in reaching the full potential of this intervention.

## Key Points

**Question:**

What is the population impact of take-home naloxone distribution at jail release to reverse opioid-related overdose among people with opioid use disorders?

**Findings:**

In this decision analytical modeling study, take-home naloxone distribution at jail release was estimated to reduce opioid-related overdose mortality. The presence of willing and capable bystanders at an opioid overdose event was a factor in program effectiveness.

**Meaning:**

Findings of this study suggest that naloxone distribution at jail release is associated with a reduction in opioid-related overdose mortality.

## Teaser

This decision analytical modeling study estimates the potential averted deaths from opioid overdose associated with distribution of take-home naloxone at jail release.

## Introduction

Opioid-related overdoses account for almost 80 000 US deaths annually.^1,2^ Persons living with opioid use disorder (OUD) who exit carceral settings are at particularly high risk. Despite evidence that medications for opioid use disorder (MOUD) are associated with reduced overdose and are cost-effective in carceral settings,^3^ the majority of US jails do not offer such treatment.^56^ Many persons reinitiate opioid use at jail release and have lower tolerance due to lack of MOUD access and forced abstinence alongside limited access to harm reduction resources during incarceration.^4,5,6,7^

When used correctly, naloxone (Narcan; Emergent) reduces the probability of fatal opioid-related overdose by at least 80%.^8,9,10^ Recent studies correspondingly emphasize the importance, feasibility, and likely economy of post–jail release naloxone distribution for decreasing overdose mortality.^1,2,5,11,12,13^

Ethical precepts constrain randomized usual-care trials to evaluate proven lifesaving interventions to address this severe mortality risk.^14^ Moreover, the outcomes of evaluated interventions reflect myriad implementation factors, mediating pathways, and contextual moderators. Dynamic computational models thus provide valuable resources to explore implementation processes and potential outcomes of pertinent interventions.^15,16,17^ For example, Macmadu and colleagues^15^ used tools similar to the present study and found that MOUD treatment linkages at jail or prison release were associated with markedly reduced mortality. Emerging research uses advanced epidemiological tools to explore the outcome of naloxone distribution in persons at highest risk.^15,18,19,20,21,22,23^

Pitt and colleagues^24^ used a compartmental framework with an aggregated study population and found that broad naloxone distribution could have reduced US opioid-related overdose mortality by 4% between 2016 and 2020. Keane and colleagues^25^ implemented a disaggregated heterogeneous agent-based model and estimated that adding secondary social network naloxone distribution through a single site could result in 42.5% fewer overdose deaths in the community relative to baseline.

Irvine and colleagues^21^ explored related questions through stochastic Markov models. They found that naloxone distribution would be especially beneficial in addressing witness-observed overdoses within fentanyl-dominated epidemics. Zang and colleagues^23^ similarly deployed microsimulation to estimate that naloxone distribution to Rhode Island residents who inject drugs would decrease mortality among witnessed opioid-related overdoses by 25.3% annually, with a mean incremental cost of $27 312 per fatal overdose averted. Naloxone distribution in carceral settings receives less systematic attention, although analyses have identified pertinent service gaps for this population.^5,13^

Informed by a combined simulation-implementation science framework,^17,26^ we used the Justice-Community Circulation Model (JCCM) to identify the facilitators and barriers of the effectiveness of naloxone distribution. Combining domain-specific data and clinical expertise with simulation models, this decision analytical model study explored feasible programmatic outcome and 1 cost-effectiveness metric—that is, incremental costs of direct naloxone distribution per averted opioid-related overdose death associated with various distribution strategies. We used sensitivity analysis to examine how complementary investments and contextual factors magnify or undermine program effectiveness. The study objective was to ascertain the population impact of take-home naloxone (THN) distribution at jail release to reverse opioid-related overdose among people with opioid use disorders.

## Methods

The JCCM is an agent-based model developed using the Repast4Py toolkit (eAppendix 2 in Supplement 1).^28^ The JCCM seeks to capture nuanced dynamics by incorporating location-specific data on population demographics, drug use, and risk behaviors along the opioid-treatment cascade of care.^29^ By scrutinizing these local parameters, the JCCM complements other implementation science perspectives,^30^ allowing the systematic investigation of internal program factors and contextual barriers and facilitators that may affect the interventions’ population impact and cost-effectiveness. Additionally, the JCCM represents a person’s perspective as they experience events, such as jail release or naloxone administration, along with their time- and location-dependent opioid-related overdose risks. In accordance with the Common Rule, this study was exempt from ethics review and informed consent requirement because it was not human participant research. The eAppendix 1 in Supplement 1 provides the Criteria for Health Economic Quality Evaluation (CHEQUE) checklist compilation applied to this analysis.^27^

**Figure 1.**
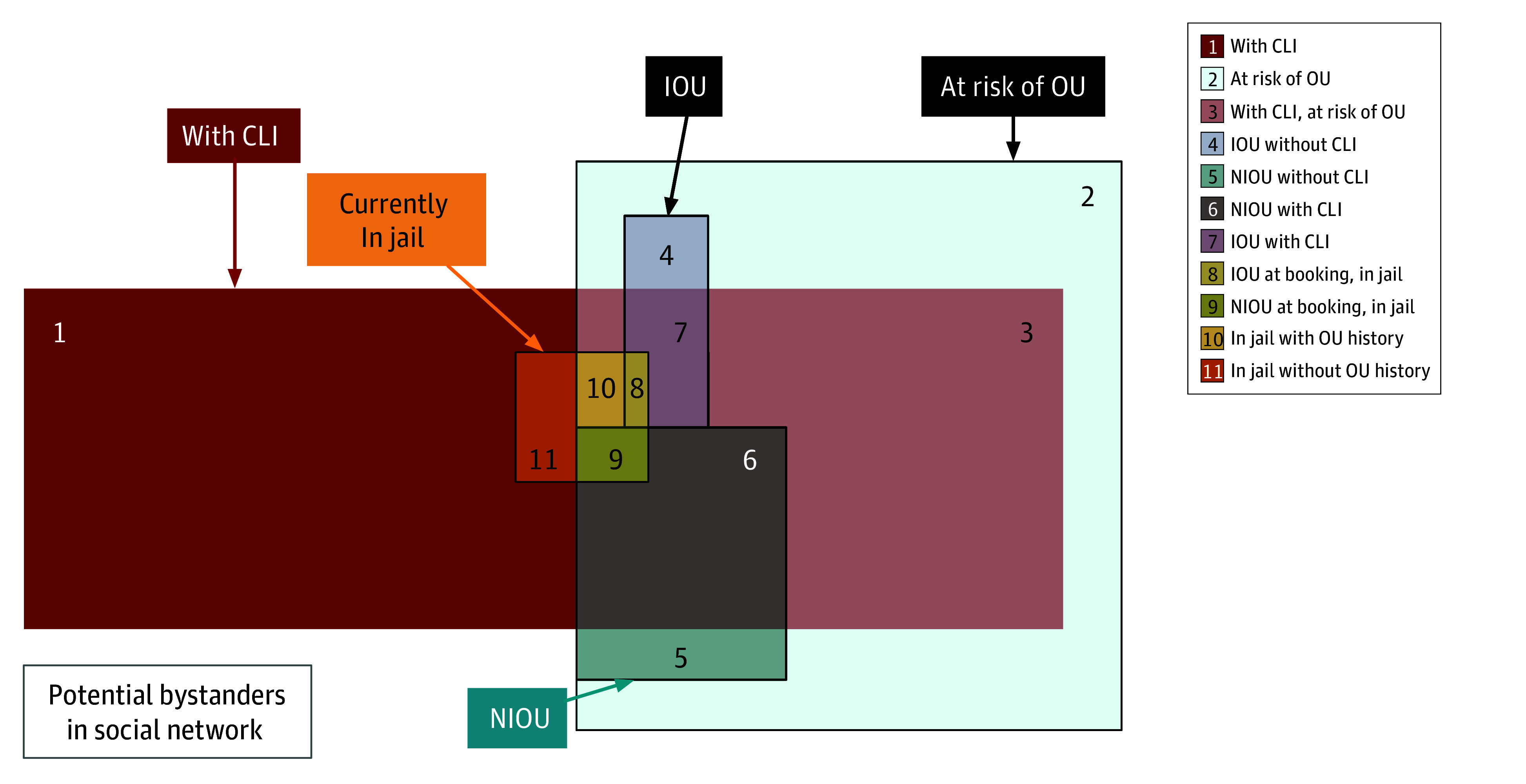
Justice-Community Circulation Model Synthetic Population Subgroups by Opioid Use and Criminal-Legal-System Involvement (CLI) CLI includes current or prior incarceration and risk of future incarceration. IOU indicates injection opioid user; NIOU, noninjection opioid use; OU, opioid user. The social network of potential bystanders at overdose events was modeled using stochastic parameter of bystander probability.

In this study, we used the JCCM to model a synthetic population of persons with and without criminal-legal-system involvement (CLI) and persons with and without illicit opioid use (Figure 1). The synthetic population aimed to capture local dynamics of persons with current or past opioid use and the locale’s total population with CLI, many of whom have not used opioids.

Persons who use and are at risk of using illicit opioids were modeled as autonomous agents. Each possessed unique demographic characteristics and time-varying states (eg, drug use and CLI). These variables were combined with risk-effect variables that together had implications for the probability of experiencing and surviving opioid-related overdose.

### Agent Characteristics

The model was based on the greater Chicago area, Cook County, Illinois, where injection^26,31^ and snorting (insufflation)^32^ have been the predominant routes of administration for nonprescription opioids. The synthetic population reflected demographic data collected from Chicago-area studies and the Chicago Department of Public Health. We derived pertinent parameters and calibrated models from multiple sources, including self-reported drug use, publicly available hospital discharge data, and opioid-related overdose deaths reported by the Cook County Medical Examiner’s Office and coroners’ offices.

The population of opioid users in Cook County, consisting of approximately 90 000 individuals, was then categorized by primary route of administration: noninjection opioid user (NIOU) and injection opioid user (IOU). For simplicity, the JCCM considered individuals to be primarily injectors or noninjectors. We modeled a population of 24 000 IOUs using published estimates^33^ and demographic data^31^ of people who inject drugs^26^ (eAppendix 3 in Supplement 1). We then estimated that the NIOU subpopulation comprised the remaining 66 000 people with noninjection routes of administration. The demographic characteristics of this subpopulation were estimated using published estimates of noninjection heroin users in Chicago and elsewhere in Cook County.^32^

### Population With History of Incarceration in Cook County Jail 

The nightly census at Cook County Jail varied, averaging approximately 6000 persons between 2018 and 2021.^34,35,36^ We posited a daily mean of 132 bookings, with mean and median stays of 54.1 and 12 days, respectively, to match published estimates (eAppendix 4 and eTable 1 in Supplement 1).^35^

The JCCM modeled a population of 50 000 persons with CLI, including those currently or previously incarcerated and those facing incarceration risks (eAppendixes 4 and 5 in Supplement 1). Demographic details for Cook County Jail–detained persons from 2016 to 2017 were obtained from the Cook County Sheriff’s website. Although the jail census has varied (recently decreasing due to the SAFE-T Act and other decarceration efforts^37^), detainee demographic characteristics have remained stable, providing opportunity to leverage the 2016-2017 demographic details to model future years.^38^ The proportions of NIOUs and IOUs with CLI were estimated from published reports of people arrested in Cook County and tested for illicit drugs (eAppendixes 5 and 6, eTable 2 in Supplement 1).^38^

### Opioid-Related Overdose and Deaths

We used the 2014 through 2021 Cook County Medical Examiner’s Office–reported opioid-related overdose death data^39^ to generate the synthetic population’s sociodemographics in 2020 (Figure 1; eTables 3 and 4 in Supplement 1) and baseline opioid-related overdoses and deaths (eTables 5 and 6 in Supplement 1). The Illinois Department of Public Health’s Opioid Data Dashboard provided opioid-related overdose and death data.^40^ Cook County yearly counts of opioid-related patient discharges were used to estimate overdose lower bounds, as these only include hospitalized individuals diagnosed with opioid-related overdose (eAppendix 7 and eTables 7 to 10 in Supplement 1). A person’s risk-adjusted opioid-related overdose probability was defined as the baseline probability, based on total opioid user population risk multiplied by the total product of all associated overdose risks specific to that person’s demographic, behavioral, or situational attributes (eAppendix 8 and eTable 11 in Supplement 1).

### Modeling Take-Home Naloxone to Individuals at Jail Release

When properly and promptly administered, THN has been estimated to lower opioid-related overdose mortality by up to 95%.^9^ THN is available in intramuscular injectable and intranasal spray formulations. Both formulations can be administered by a loved one, a peer who uses drugs, or another bystander or acquaintance, in addition to first responders.

We are not positioned to provide a full cost-effectiveness analysis, which depends on the trajectory of future health care costs and other processes beyond the JCCM. As a secondary outcome, we provided estimates of direct program costs per fatal overdose averted. We estimated an incremental cost-effectiveness ratio (ICER) from the social perspective using cost per death averted by each intervention scenario compared with no intervention. Following Behrends and colleagues,^41^ we assumed upper-bound direct program costs (in 2017 dollars) of $76 per kit of intranasal naloxone. Furthermore, we assumed that probabilities of opioid-related overdose reversal with THN are independent of dose or formulation and that individuals obtain a new THN kit immediately after use of an existing kit.

Modeled interventions included 3 complementary THN kit distribution channels: (1) community clinics, pharmacies, harm reduction practitioners, and OUD treatment facilities; (2) jail, which gives THN kits directly to persons with a history of opioid use at their release; and (3) social network or peers of persons released from incarceration. The JCCM did not explicitly model social networks, but it modeled the probability that a released person has at least 1 peer with a THN kit when opioid-related overdose occurs (eAppendixes 9 and 10 in Supplement 1).

**Figure 2.**
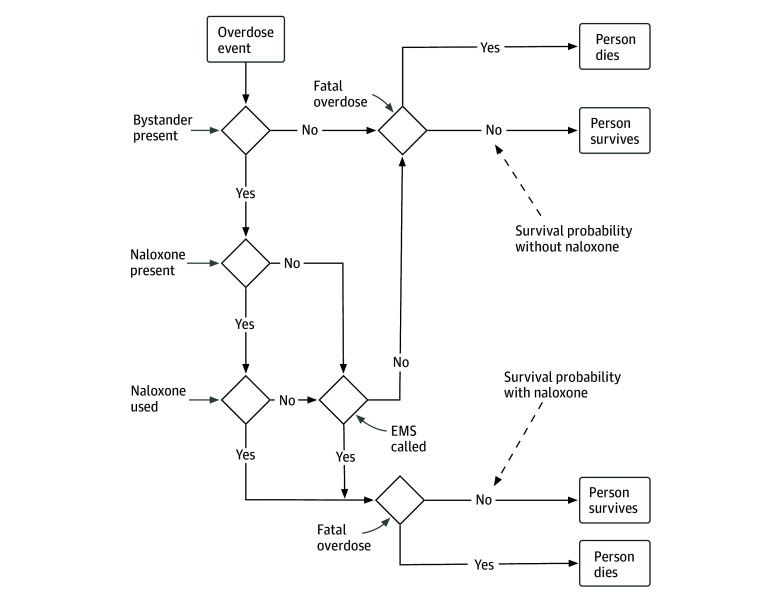
Flowchart of Bystander Behavior Logic During a Witnessed Opioid-Related Overdose Event Showing Modeled Decisions of the Bystander and Outcomes for Person Experiencing the Overdose Without a bystander, the survival probability for the person is modeled by the fatality probability per overdose. When a bystander is present and willing to intervene, the bystander may choose to call emergency medical services (EMS) and administer naloxone if available. The bystander logic separately considers the probability of naloxone availability at the overdose location and the probability that it is actually or effectively used.

THN is effective only when a bystander is present, willing, and able to intervene. We varied the probability of bystander presence from 30% to 90% (Table 1).^8,10^ Figure 2 shows the possible sequence of JCCM events following overdose events. Bystanders were not explicitly modeled as agents; however, potential bystander actions during otherwise-fatal opioid-related overdoses were posited to alter survival probability.

**Table 1.** Input Parameters in the Sensitivity Analysis^a^

JCCM parameter	Estimate^b^	
	Lower bound	Upper bound
Nonintervention parameters		
Percentage of jail inmates who use opioids primarily through injection (IOU)	2.0	5.0
Percentage of jail inmates who use opioids primarily through noninjection (NIOU)	6.0	15.0
Baseline daily overdose probability per person, %	0.016	0.050
Fatal probability per overdose, %	7.0	13.0
RR of overdose		
Female	0.21	1.41
Male	1 [Reference]	1 [Reference]
IOU	0.50	9.00
NIOU	1.0 [Reference]	1.0 [Reference]
Jail release wk 0-2	2.00	10.0
Jail release wk 3-4	2.00	6.00
Jail release wk ≥5	1.00	2.00
No previous jail incarceration	1 [Reference]	1 [Reference]
Age 18-24 y	1 [Reference]	1 [Reference]
Age 25-29 y	0.39	3.78
Age ≥30 y	0.72	5.15
Probability of a bystander present during overdose, %	30.0	90.0
Probability of administering naloxone during overdose, %	50.0	90.0
Probability of bystander calling EMS during overdose, %	20.0	80.0
Intervention parameters		
Naloxone distribution from jail to individuals released from jail	0	100.0
Naloxone distribution from community channels to the OU population	0	30.0
Naloxone distribution from the social network of individuals released from jail	0	30.0

Abbreviations: EMS, emergency medical services; IOU, injection opioid user; JCCM, Justice-Community Circulation Model; NIOU, noninjection opioid user; OU, opioid user; RR, relative risk.

^a^ Data from eAppendixes 4, 5, and 7; eTable 2; and eTables 5 to 11 in Supplement 1.

^b^ Opioid-related overdose baseline probability and overdose risk multipliers; include lower and upper estimates used to bound the overdose risk parameters in the sensitivity analysis, with data based on estimates for Cook County, Illinois, in 2020. Risk factors for opioid-related overdose are generally reported for fatal overdoses as univariate or adjusted hazard ratios, risk ratios, and odds ratios. In the JCCM, these hazards are considered to affect the probability of any opioid-related overdose because it is not possible to decouple risks for fatal and nonfatal overdoses using the published data.

### Sensitivity Analysis

The sensitivity analysis had 3 interrelated goals: (1) to understand programmatic implications of point-estimate parameter uncertainties, (2) to identify parameters important to intervention effectiveness, and (3) to scrutinize the contextual factors associated with enhanced or constrained population impact of proposed interventions. We considered variation in the following outcomes: (1) estimated annual overdoses and deaths in the total opioid use population, (2) annual overdoses and deaths among individuals released from jail, (3) opioid-related overdose deaths averted compared with baseline without intervention or compared with less-intensive interventions, (4) the number needed to treat to prevent 1 overdose death compared with baseline without intervention, and (5) the secondary metric of direct program costs per overdose death averted.

### Statistical Analysis

Model inputs included the 2020 estimated parameters of the THN intervention itself as well as contextual parameters, such as annual opioid-related overdose risks, or the probability of bystander presence when overdose occurs (Table 1). We performed a global sensitivity analysis to ascertain the total contribution of each input parameter to the variance in model output parameters (eAppendix 11 and eTable 12 in Supplement 1). Initial sensitivity analysis provides a useful screen to identify and study the subset of model inputs that change modeled outcomes the most (eTable 13 in Supplement 1). We performed parameter screening using the Sobol method to estimate relative contributions of each input parameter.^42^ Simulation scenarios were run over 365 days, with a time step of 1 day. A 90-day warm-up period preceded the simulated 365 days to achieve steady state population dynamics. We examined 27 different scenarios of high, medium, and low levels of THN kit distribution across community, jail, and social network, along with a baseline scenario (scenario 1) without THN kit distribution (eTable 14 in Supplement 1). Data analysis was performed between January 2022 and March 2024 using R, version 4.3 (R Project for Statistical Computing).

## Results

### Implications of THN Distribution for Overdose Mortality in the Overall Population

**Figure 3.**
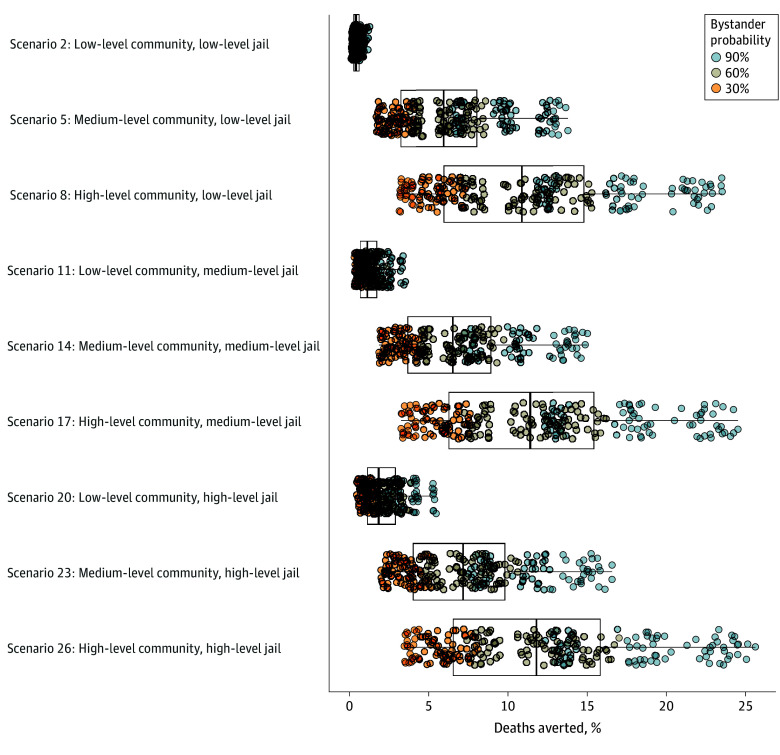
Percentage of Opioid-Related Overdose Deaths Averted for the Selected Community and Jail Naloxone Distribution Scenarios Each circle indicates a unique combination of nonintervention parameters averaged over 10 stochastic runs, and each color indicates the value of bystander probability for the individual run. Right and left sides of the boxes represent the 25th and 75th percentiles, respectively; the vertical line inside boxes represents the median; and whiskers represent minimum and maximum values.

Community distribution of THN kits had the largest projected implications for averting overdose deaths within the overall opioid user population, a median (IQR) decrease of 11.70% (6.57%-15.75%). Within a given level of community THN distribution, increasing jail distribution also increased deaths averted (Figure 3, Table 2). Naloxone interventions with the highest median percentage of deaths averted corresponded to the highest level (30%) of community distribution (scenarios 7-9, 16-18, 25-27). For example, in scenarios 25 to 27, the median (IQR) percentage of deaths averted was 11.70% (6.57%-15.75%) (Table 2). Interventions with jail distribution only, but no community distribution, resulted in fewest averted deaths (scenarios 1-3, 10-12, and 19-21). For example, in scenarios 19 to 21, the median (IQR) percentage of deaths averted was 1.79% (1.09%-2.82%) (Table 2).

**Table 2.** Take-Home Naloxone Distribution in the JCCM

THN distribution scenario	THN distribution channel, %			Projected median (IQR)^a^			
	Community	Jail release	Social network	THN kits distributed, No.^b^	Percentage of deaths averted^c^	Cost per death averted, 2017 $^d^	NNT^e^
1	0	0	0	0	0	NA	NA
2	0	0	15	958 (710-1188)	0.34 (0.20-0.56)	9100 (5900-14 700)	119.4 (77.8-193.0)
3	0	0	30	1909 (1401-2368)	0.67 (0.41-1.09)	9000 (6200-14 100)	118.1 (81.3-185.9)
4	15	0	0	15 297 (14 535-16 353)	5.58 (3.08-7.72)	9800 (6500-15 300)	129.0 (85.0-201.1)
5	15	0	15	16 193 (15 487-17 362)	5.86 (3.28-8.16)	9900 (6500-15 100)	130.3 (85.8-198.9)
6	15	0	30	17 222 (16 377-18 303)	6.10 (3.44-8.55)	9900 (6600-15 400)	130.5 (86.4-202.7)
7	30	0	0	30 555 (29 041-32 590)	10.60 (5.83-14.49)^f^	10 000 (6700-15 500)	131.9 (87.5-204.3)
8	30	0	15	31 494 (30 111-33 662)	10.80 (5.99-14.68)^f^	10 100 (6700-15 600)	132.8 (87.8-205.4)
9	30	0	30	32 285 (30 933-34 551)	11.00 (6.10-14.93)^f^	10 200 (6700-15 800)	133.9 (88.5-207.6)
10	0	50	0	3095 (2252-3864)	0.89 (0.54-1.41)	11 100 (7500-17 200)	145.6 (98.8-226.0)
11	0	50	15	3994 (2890-4989)	1.10 (0.65-1.70)	11 900 (8000-18 400)	156.8 (105.7-241.5)
12	0	50	30	4880 (3520-6114)	1.25 (0.74-1.94)	12 800 (8400-19 500)	168.8 (111.0-256.5)
13	15	50	0	18 405 (17 437-19 613)	6.38 (3.55-8.80)	10 300 (6800-15 900)	135.0 (89.0-208.7)
14	15	50	15	19 356 (18 197-20 686)	6.50 (3.66-8.94)	10 500 (6900-16 300)	137.9 (90.7-213.9)
15	15	50	30	20 283 (18 948-21 646)	6.63 (3.72-9.18)	10 700 (7100-16 500)	140.5 (93.4-217.5)
16	30	50	0	33 533 (32 015-35 573)	11.00 (6.23-15.16)^f^	10 400 (6900-16 000)	136.6 (90.4-210.8)
17	30	50	15	34 641 (32 932-36 608)	11.40 (6.25-15.27)^f^	10 500 (7000-16 300)	138.8 (91.5-214.7)
18	30	50	30	35 563 (33 687-37 603)	11.40 (6.36-15.44)^f^	10 700 (7100-16 500)	140.9 (93.5-217.1)
19	0	100	0	6189 (4502-7709)	1.79 (1.09-2.82)	11 000 (7500-16 800)	144.1 (98.7-221.6)
20	0	100	15	7026 (5077-8792)	1.79 (1.09-2.82)	12 500 (8400-19 200)	163.8 (111.1-252.1)
21	0	100	30	7855 (5653-9871)	1.79 (1.09-2.82)	13 900 (9400-21 400)	183.1 (123.6-282.0)
22	15	100	0	21 672 (19 977-23 130)	7.07 (4.01-9.92)	10 500 (7000-16 400)	138.6 (91.7-215.3)
23	15	100	15	22 536 (20 764-24 056)	7.07 (4.01-9.92)	10 900 (7200-17 100)	143.6 (95.2-225.0)
24	15	100	30	23 407 (21 566-25 047)	7.07 (4.01-9.92)	11 300 (7500-17 700)	149.2 (98.7-232.6)
25	30	100	0	36 642 (34 803-39 017)	11.70 (6.57-15.75)^f^	10 700 (7100-16 500)	141.2 (92.8-217.7)
26	30	100	15	37 563 (35 551-40 074)	11.70 (6.57-15.75)^f^	10 900 (7200-16 900)	143.9 (94.9-222.9)
27	30	100	30	38 503 (36 265-40 995)	11.70 (6.57-15.75)^f^	11 200 (7400-17 300)	147.4 (96.9-227.8)

Abbreviations: JCCM, Justice-Community Circulation Model; NA, not applicable; NNT, number needed to treat; THN, take-home naloxone.

^a^ Projected median (IQR) values for the entire opioid user population in the JCCM, for 2020 in Cook County, Illinois.

^b^ THN kits distributed included kits provided to both opioid users in the community and those directly at jail release or to the social network of persons released from jail.

^c^ Deaths averted included deaths in the entire opioid use population in the JCCM, including individuals with criminal-legal-system involvement.

^d^ Cost per death averted is the total intervention cost (community + jail + jail social network) divided by the number of deaths averted in the entire opioid use population in the JCCM.

^e^ The NNT to prevent 1 opioid-related overdose death is the number of THN kits distributed divided by the number of overdose deaths averted compared with the baseline scenario without naloxone (scenario 1).

^f^ Scenarios with the most overdoses averted compared with the baseline scenario without naloxone (scenario 1).

Across all 27 scenarios, increased probability of bystander presence increased the percentage of deaths averted (Figure 3). For interventions with the highest combined levels of community and jail distribution, the median percentage of deaths averted in the overall opioid user population (11.7%) (Figure 3, Table 2) increased to 25.0% when the probability of an active bystander reached 90.0% (scenario 26; Figure 3). The probability of bystander presence at an opioid overdose showed the greatest proportional contribution (27.15%) to the variance in deaths averted in persons released from jail (eTable 12 in Supplement 1).

### Implications of THN Distribution for Overdose Mortality in a Population Released From Jail 

We also examined averted deaths among individuals released from jail in 2020 in Cook County, modeling the 27 distribution scenarios. Targeted jail THN distribution produced the highest median (IQR) percentage of averted deaths in this population (24.4% [15.7%-33.6%]) (eFigure 1 in Supplement 1). Increasing THN kit distribution to both the community and to persons released from jail resulted in proportionally increased averted deaths among people released from jail, with more targeted jail-based interventions having a larger proportion of averted deaths within this group (eFigures 2 to 4 in Supplement 1). THN interventions exclusively for persons released from jail (scenarios 19-27) resulted in the largest median (IQR) percentage of deaths averted in this specific group, from 23.3% (14.7%-32.5%) to 24.4% (15.7%-33.6%).

The high percentage of deaths averted specifically in the jail distribution group (eFigure 1 in Supplement 1) reflects the unique prevention opportunity presented at jail release. High probability of a present bystander (90.0%) consistently reduced overdose deaths, with a maximum of 48.9% of deaths averted in some simulations.

### Direct Program Costs and Associated Incremental Cost-Effectiveness Metrics Per Averted Death

Across the 27 distribution scenarios, we found low variance in direct program costs per averted fatal overdose and in the number needed to treat for the overall opioid user population (Table 2). The median (IQR) cost per death averted ranged from $9000 ($6200-$14 100) to $13 900 ($9400-$21 400) across modeled scenarios. An ICER was then estimated using the cost per death averted (Table 2), with each scenario cost compared with the no-intervention scenario 1 and the deaths averted in each scenario as the ICER denominator.

Scenarios 2 and 3, which focused on THN kit distribution to the social network of the person released from jail, displayed the lowest median (IQR) costs per death averted ($9100 [$5900-$14 700] and $9000 [$6200-$14 100], respectively). However, scenarios 2 and 3 also resulted in the lowest deaths averted; for example, scenario 2 showed only a median (IQR) percentage of 0.34% (0.20%-0.56%). Scenarios with the highest number of averted deaths (eg, scenarios 25-27) resulted in only marginal increases in median (IQR) costs per averted death ($10 700 [$7100-$16 500] to $11 200 [$7400-$17 300]) compared with the lowest cost scenarios (eg, scenario 2). Costs per averted death were linear as the intensity of intervention (as measured by the number of THN kits distributed) was increased (Table 2; eFigures 5 and 6 in Supplement 1).

## Discussion

Our findings underscored the effectiveness and economy of THN distribution to save lives. Estimated direct program costs per averted fatal overdose were low (ranged from $9000 to $13 900), particularly compared with standard cost-effectiveness thresholds ($6-12 million per averted death) used to evaluate criminal justice and occupational safety interventions.^43^ A combination approach that provided THN kits within the community, at jail release, and to peers of individuals with OUD recently released from jail saved the most lives.

The results also underscored the importance of contextual factors. One such barrier emerges when the institutional structures of jails and prisons come into conflict with harm-reduction services provided to formerly incarcerated persons.^44^ Implementation analyses by Showalter and colleagues^45^ described how such barriers might be addressed. Mobilizing district attorneys and other law enforcement officials can support interorganizational bridges that facilitate naloxone distribution. Jail-based health professionals can be internal champions for harm reduction interventions.^5^

This study identified the presence of a properly equipped, naloxone-trained peer or bystander as a key focus for intervention. Such a finding highlights the need to reduce the proportion of people who use drugs alone and the value of public health messaging to support THN deployment and to train potential bystanders in proficient THN use. Additionally, our findings suggest the need to prioritize implementation trials^46^ of interventions to promote reliable naloxone possession and administration among people who use opioids and those positioned to intervene in situations where overdose is most likely to occur.^47^ Implementation trials could include specific analyses of implementation costs^48^ as well as investigation of innovative virtual and telephone-based measures to protect persons who might otherwise use drugs alone.^46,49^

We presumed willingness to carry and receive THN as well as attention to the tactile realities that confront people who use drugs. In qualitative interviews, most opioid users reported having some naloxone training but no presence of naloxone at recent overdose events.^50^ People who inject drugs commonly choose not to carry THN due to fear of the legal consequences from being observed with THN, substance use stigma, and fear of harming or traumatizing someone through medically unnecessary administration.^51^

Stigmatizing frames that identify naloxone with continued substance use, along with punitive organizational policies toward naloxone in shelters and other settings, may further deter naloxone possession, thus hindering effective emergency use. Additionally, care must be taken during efforts such as homeless encampment sweeps, not to increase overdose fatality by reducing the availability of THN-equipped bystanders when opioid-related overdose occurs.^52^

Bowles and colleagues^53^ underscored the importance of identity-competent messaging to address naloxone refusal among ambivalent, newly abstinent persons, who may identify naloxone with continued drug use. Messages that emphasize THN carrying as an opportunity to provide lifesaving aid to others may be especially respectful and effective. Acknowledging the inherent limitations of public health messaging to address structural challenges, messaging analyses have also identified strategies to disseminate destigmatizing information regarding the value of THN availability and access as well as proper use. These studies provide a valuable reminder that harm reduction is implemented within a social and epidemiological context. Its outcome is correspondingly affected by a web of complementary interventions, legal and social practices, epidemiological vulnerabilities, peer relationships, and support networks.

Agent-based modeling provides a valuable approach to explore these linkages and to improve implementation. Complementing empirical program evaluation and ethnographic and qualitative research informed by people who use drugs, these agent-based modeling methods identify critical program and contextual factors that can alter outcomes and program performance. Sensitivity analyses offer a particularly useful guidance. Understanding the critical internal and external factors, in turn, informs the practical operation of feasible interventions.

### Limitations

This study should be interpreted in light of several limitations. The JCCM reflects a complex aggregation of domain-specific expertise along with data obtained from the research literature and from state and municipal sources (eAppendix 12 in Supplement 1 provides details). Our framework could be enriched to include greater programmatic realism to engage other aspects of program quality and acceptability for people who use drugs. We did not consider whether bystanders must administer multiple naloxone doses,^54^ nor did we model variance in emergency medical services response time. We did not directly engage post–COVID-19 variability in correctional practices, many of which induced lower jail populations that were weighted toward more serious offenders. Policy analysts have called for resumption of the Arrestee Drug Abuse Monitoring Program and similar efforts to improve epidemiological surveillance of drug-use patterns in carceral populations and others at risk for opioid-related overdose.^55^

We did not perform a full cohort simulation of all costs and quality of life. Thus, we did not address the full range of future benefits and costs associated with individual survival. We computed direct costs of THN kit provision per averted death over the 1-year simulation period, rather than the present discounted cost of the full-service bundle per quality-adjusted life-year over a long time horizon that could be examined within an elaborate cost-utility analysis. An important finding is that direct program costs per prevented opioid-related overdose death were low (consistently less than $23 000 across the THN distribution scenarios), which is consistent with findings in prior studies of naloxone distribution.^23^

## Conclusions

This decision analytical modeling study found that THN distribution at jail release is an economical and feasible approach to slow the opioid-related overdose epidemic. Training and preparation of proficient and willing bystanders are central factors in fulfilling the potential of this intervention.

## Notes

Supplement 1. **eAppendix 1.** Online CHEQUE Tool (2023 Version) **eAppendix 2.** Justice-Community Circulation Model **eAppendix 3.** Model Opioid Use Population Estimates **eAppendix 4.** Cook County Jail Population Estimates **eTable 1.** Summary Statistics for the JCCM Generated Length of Incarceration Distribution (Days) **eAppendix 5.** Opioid Use Among Arrestees **eTable 2.** JCCM Input Values for the Model Population Sizes **eAppendix 6.** JCCM Synthetic Population Generation **eTable 3.** A Sample Generated JCCM Non-CLI Population Showing the Numbers of Individuals for Non-Injection Opioid Users (NIOU) and Injection Opioid Users (IOU) **eTable 4.** A Sample Generated JCCM CLI Population of 50,000 Individuals Showing the Numbers of Individuals for Non-Injection Opioid Users (NIOU) and Injection Opioid Users (IOU), Assuming a Fraction of CLI Individuals Who Are IOU of 3.5% and a Fraction of CLI Individuals Who Are NIOU of 10% **eAppendix 7.** Estimating Opioid-Related Overdose and Deaths **eTable 5.** Yearly Opioid-Related Overdose Deaths (OODs) in Illinois State and Cook County, IL **eTable 6.** Data Used to Estimate Yearly Non-Fatal Opioid Overdose Events in Cook County, IL **eTable 7.** Estimated Ranges of Yearly Fatal and Non-Fatal Opioid Overdoses for Cook Co., IL **eTable 8.** JCCM Input Values for Overdose Rates and Probabilities for Simulation Year 2018 **eTable 9.** JCCM Input Values for Overdose Rates and Probabilities for Simulation Year 2020 **eTable 10.** Nominal Non-Fatal and Fatal Overdose Probabilities per Year in Cook Co. IL **eAppendix 8.** Variables That Affect Overdose Risks and Survivals **eTable 11.** Model Risk Factors That Affect Overdose Probability **eAppendix 9.** Naloxone Intervention Variables **eAppendix 10.** Model Agent Behavior Logic **eAppendix 11.** Model Software Implementation and Model Parameter Screening **eTable 12.** Sobol Total Sensitivity Indices for Deaths Averted for the Entire Model OU Population, and Deaths Averted for the Population of Persons Released From Jail, Ranked by Number of Deaths Averted in the Released Persons Group **eTable 13.** Non-Intervention Parameters Values for Intervention Scenarios Analysis **eTable 14.** Intervention Parameter Values for Intervention Scenarios Analysis **eAppendix 12.** Assumptions and Limitations of the JCCM **eFigure 1.** Deaths Averted for the Jail Release Group for Selected Naloxone Intervention Scenarios That Focus on the Effects of the Community Level and Jail Level Naloxone Distribution **eFigure 2.** Percent of Opioid-Related Overdose Deaths Averted for the Entire OU Population for Selected Naloxone Intervention Scenarios **eFigure 3.** Percent of Opioid-Related Overdose Deaths Averted for the Jail Release Group (Bottom) for Selected Naloxone Intervention Scenarios **eFigure 4.** Scatterplot Showing the Relationship Between the Percent of Deaths Averted in the Entire OU Model Population and the Number of Overdoses Witnessed and the Number of Naloxone Kits Distributed **eFigure 5.** Median Cost ($1000s) vs Deaths Averted for Each of the 27 Simulated Scenarios as Individual Points, Styled by the Level of Naloxone Distributed via Jail Release Jail and Jail Social Networks (0%, 15%, 30%), and Community Level Naloxone Distribution 0% (bottom), 15% (middle), and 30% (top) **eFigure 6.** Median Cost ($1000s) vs Deaths Averted for Each of the 27 Simulated Scenarios as Individual Points, Styled by the Level of Naloxone Distributed via Jail Release Jail and Jail Social Networks (0%, 15%, 30%), and Community Level Naloxone Distribution 0% (left), 15% (middle), and 30% (right)
eReferences

Supplement 2.
Data Sharing Statement
