# Supplementary material for: Agent-Based Model of Combined Community- and Jail-Based Take-Home Naloxone Distribution
Source: JAMA Netw Open. 2024 Dec 10;7(12):e2448732. doi: 10.1001/jamanetworkopen.2024.48732 (PMC11632540; doi:10.1001/jamanetworkopen.2024.48732)
Supplement: Supplement 1. — eAppendix 1. Online CHEQUE Tool (2023 Version) eAppendix 2. Justice-Community Circulation Model eAppendix 3. Model Opioid Use Population Estimates eAppendix 4. Cook County Jail Population Estimates eTable 1. Summary Statistics for the JCCM Generated Length of Incarceration Distribution (Days) eAppendix 5. Opioid Use Among Arrestees eTable 2. JCCM Input Values for the Model Population Sizes eAppendix 6. JCCM Synthetic Population Generation eTable 3. A Sample Generated JCCM Non-CLI Population Showing the Numbers of Individuals for Non-Injection Opioid Users (NIOU) and Injection Opioid Users (IOU) eTable 4. A Sample Generated JCCM CLI Population of 50,000 Individuals Showing the Numbers of Individuals for Non-Injection Opioid Users (NIOU) and Injection Opioid Users (IOU), Assuming a Fraction of CLI Individuals Who Are IOU of 3.5% and a Fraction of CLI Individuals Who Are NIOU of 10% eAppendix 7. Estimating Opioid-Related Overdose and Deaths eTable 5. Yearly Opioid-Related Overdose Deaths (OODs) in Illinois State and Cook County, IL eTable 6. Data Used to Estimate Yearly Non-Fatal Opioid Overdose Events in Cook County, IL eTable 7. Estimated Ranges of Yearly Fatal and Non-Fatal Opioid Overdoses for Cook Co., IL eTable 8. JCCM Input Values for Overdose Rates and Probabilities for Simulation Year 2018 eTable 9. JCCM Input Values for Overdose Rates and Probabilities for Simulation Year 2020 eTable 10. Nominal Non-Fatal and Fatal Overdose Probabilities per Year in Cook Co. IL eAppendix 8. Variables That Affect Overdose Risks and Survivals eTable 11. Model Risk Factors That Affect Overdose Probability eAppendix 9. Naloxone Intervention Variables eAppendix 10. Model Agent Behavior Logic eAppendix 11. Model Software Implementation and Model Parameter Screening eTable 12. Sobol Total Sensitivity Indices for Deaths Averted for the Entire Model OU Population, and Deaths Averted for the Population of Persons Released From Jail, Ranked by Number of Deaths Averted in the Released Pe [file jamanetwopen-e2448732-s001.pdf]

Supplemental Online Content

Tatara E, Ozik J, Pollack HA, et al. Agent-based model of combined community- and jail-based take-home naloxone distribution. *JAMA Netw Open*. 2024;7(12):e2448732. doi:10.1001/jamanetworkopen.2024.48732

eAppendix 1. Online CHEQUE Tool (2023 Version)
eAppendix 2. Justice-Community Circulation Model
eAppendix 3. Model Opioid Use Population Estimates
eAppendix 4. Cook County Jail Population Estimates
eTable 1. Summary Statistics for the JCCM Generated Length of Incarceration Distribution (Days)
eAppendix 5. Opioid Use Among Arrestees
eTable 2. JCCM Input Values for the Model Population Sizes
eAppendix 6. JCCM Synthetic Population Generation
eTable 3. A Sample Generated JCCM Non-CLI Population Showing the Numbers of Individuals for Non-Injection Opioid Users (NIOU) and Injection Opioid Users (IOU)
eTable 4. A Sample Generated JCCM CLI Population of 50,000 Individuals Showing the Numbers of Individuals for Non-Injection Opioid Users (NIOU) and Injection Opioid Users (IOU), Assuming a Fraction of CLI Individuals Who Are IOU of 3.5% and a Fraction of CLI Individuals Who Are NIOU of 10%
eAppendix 7. Estimating Opioid-Related Overdose and Deaths
eTable 5. Yearly Opioid-Related Overdose Deaths (OODs) in Illinois State and Cook County, IL
eTable 6. Data Used to Estimate Yearly Non-Fatal Opioid Overdose Events in Cook County, IL
eTable 7. Estimated Ranges of Yearly Fatal and Non-Fatal Opioid Overdoses for Cook Co., IL
eTable 8. JCCM Input Values for Overdose Rates and Probabilities for Simulation Year 2018
eTable 9. JCCM Input Values for Overdose Rates and Probabilities for Simulation Year 2020
eTable 10. Nominal Non-Fatal and Fatal Overdose Probabilities per Year in Cook Co. IL
eAppendix 8. Variables That Affect Overdose Risks and Survivals
eTable 11. Model Risk Factors That Affect Overdose Probability
eAppendix 9. Naloxone Intervention Variables
eAppendix 10. Model Agent Behavior Logic
eAppendix 11. Model Software Implementation and Model Parameter Screening
eTable 12. Sobol Total Sensitivity Indices for Deaths Averted for the Entire Model OU Population, and Deaths Averted for the Population of Persons Released From Jail, Ranked by Number of Deaths Averted in the Released Persons Group
eTable 13. Non-Intervention Parameters Values for Intervention Scenarios Analysis
eTable 14. Intervention Parameter Values for Intervention Scenarios Analysis
eAppendix 12. Assumptions and Limitations of the JCCM
eFigure 1. Deaths Averted for the Jail Release Group for Selected Naloxone Intervention Scenarios That Focus on the Effects of the Community Level and Jail Level Naloxone Distribution
eFigure 2. Percent of Opioid-Related Overdose Deaths Averted for the Entire OU Population for Selected Naloxone Intervention Scenarios
eFigure 3. Percent of Opioid-Related Overdose Deaths Averted for the Jail Release Group (Bottom) for Selected Naloxone Intervention Scenarios

eFigure 4. Scatterplot Showing the Relationship Between the Percent of Deaths Averted in the Entire OU Model Population and the Number of Overdoses Witnessed and the Number of Naloxone Kits Distributed
eFigure 5. Median Cost ($1000s) vs Deaths Averted for Each of the 27 Simulated Scenarios as Individual Points, Styled by the Level of Naloxone Distributed via Jail Release Jail and Jail Social Networks (0%, 15%, 30%), and Community Level Naloxone Distribution 0% (bottom), 15% (middle), and 30% (top)
eFigure 6. Median Cost ($1000s) vs Deaths Averted for Each of the 27 Simulated Scenarios as Individual Points, Styled by the Level of Naloxone Distributed via Jail Release Jail and Jail Social Networks (0%, 15%, 30%), and Community Level Naloxone Distribution 0% (left), 15% (middle), and 30% (right)
eReferences

This supplemental material has been provided by the authors to give readers additional information about their work.

© 2024 Tatara E et al. *JAMA Network Open*

eAppendix 1. Online CHEQUE Tool (2023 Version)

We thank you for your time spent taking this survey.
Your response has been recorded.

The Criteria for Health Economic Quality Evaluation (CHEQUE) Tool

The peer-reviewed manuscript on the development of the CHEQUE tool is available HERE.

Cost-effectiveness analysis (CEA) has played an increasingly important role in clinical guidelines and coverage/reimbursement decisions. As the number of published CEAs continues to grow, there is a substantial need for assessing the quality of and comparability across cost-effectiveness evidence. However, despite multiple checklists and guidelines, practical tools for differentiating the methodological and reporting quality of CEAs are lacking.

The CHEQUE tool attempts to fill this need by providing a web-based, user-friendly numerical grading system that measures the methodological and reporting quality of CEAs separately. The primary audiences for the CHEQUE tool include researchers conducting CEAs, decision-makers considering cost-effectiveness evidence, and editors assessing CEAs for publication.

The CHEQUE tool includes 48 quality attributes (24 attributes that assess methodological quality plus 24 that assess reporting quality). Each attribute is assigned a maximum score ranging from 1 to 9 based on the attribute's relative importance.

To use the online CHEQUE tool, reviewers select their level of agreement with each quality attribute: "Yes", "Somewhat", "No", or "Not Applicable". Each level corresponds to different weights for calculating the final score: Yes=full credit (i.e., the assigned importance score is multiplied by 1.0), Somewhat=half credit (i.e., the assigned importance score is multiplied by 0.5), No=no credit. The reviewers can determine how to deal with "Not Applicable" in the next section.

© 2024 Tatara E et al. *JAMA Network Open*

After completing the assessment, the online CHEQUE tool will automatically calculate final scores for methodological quality and reporting quality. Reviewers can download their assessment results in a PDF format.

Please let us know if you have any suggestions or questions.

Principal Investigator: David D. Kim, PhD (ddk@uchicago.edu), University of Chicago
Project Team: Lauren Do, Patty Synnott, Tara Lavelle, Lisa Prosser, John Wong, and Peter Neumann

© 2024 Tatara E et al. *JAMA Network Open*

Study Title

An Agent-based model of combined community and jail-based take-home naloxone distribution

The final CHEQUE tool quality scores can be calculated using two different methods.

Please select your preferred scoring method.

Scoring Method 1: Assign Full Credit to "Not Applicable"	Scoring Method 2: Exclude "Not Applicable"	Scoring Method 1 & 2
Quality attributes deemed "Not Applicable" will be given the full credit. (100 total possible points for each methodological and reporing quality)	Quality attributes deemed "Not Applicable" will be excluded from the final score. (The total possible points will vary depending on the number of attributes selected "Not Applicable")	Receive both sets of scores.
○	○	●

Decision Problem and Scope

Please select your level of agreement for each attribute.

	Yes	Somewhat	No	Not Applicable	Comments
M1. The analysis answers an important question for decision-making.	●	○	○	○	
M2. The study objective (decision problem) is measurable.	●	○	○	○	
R1. The study objectives (or decision problems) are clearly stated.	●	○	○	○	

Intervention and Comparator(s)

Please select your level of agreement for each attribute.

	Yes	Somewhat	No	Not Applicable	Comments
M3. Comparator is the best possible option that appropriately measures the opportunity cost of using the new treatment.	○	●	○	○	
R2. All aspects of the interventions that may affect their cost-effectiveness are defined (e.g., frequency of delivery, setting of delivery, specific technologies used).	○	○	●	○	
R3. Comparator(s) are clearly stated	●	○	○	○	

© 2024 Tatara E et al. *JAMA Network Open*

Perspective

	Please select your level of agreement for each attribute.				Comments
	Yes	Somewhat	No	Not Applicable	
M4. Analytic perspective is appropriate to answer the research question posed.	X				
R4. Perspective is clearly stated.	X				

Population

	Please select your level of agreement for each attribute.				Comments
	Yes	Somewhat	No	Not Applicable	
M5. The scope of the study encompasses all populations affected by the intervention.	X				
R5. Target population is clearly stated.	X				

Outcome Measures

	Please select your level of agreement for each attribute.				Comments
	Yes	Somewhat	No	Not Applicable	
M6. Health outcomes are measured in health metrics that aggregate survival and health-related quality-of-life or disability (e.g., QALY or DALY).			X		
R6. Primary outcome measure(s) are clearly stated.	X				
R7. Incremental cost-effectiveness ratios (ICERs) are reported.		X			

Time Horizon

	Please select your level of agreement for each attribute.				Comments
	Yes	Somewhat	No	Not Applicable	
M7. Time horizon is sufficient to reflect all important differences between intervention(s) and comparator(s).		X			
R8. Time horizon is clearly stated.	X				

© 2024 Tatara E et al. *JAMA Network Open*

**Discounting**	Please select your level of agreement for each attribute.				Comments
	Yes	Somewhat	No	Not Applicable	
M8. Costs and health effects that occur in the future are discounted to their present value using a recommended discount rate.	○	○	○	●	
R9. Discount rate is clearly stated.	○	○	○	●	

**Modeling**	Please select your level of agreement for each attribute.				Comments
	Yes	Somewhat	No	Not Applicable	
M9. The chosen model type is appropriate to address study questions.	●	○	○	○	
M10. Structure of the model reflects the underlying health condition and the impact of interventions.	●	○	○	○	
M11. Modeling assumptions are reasonable given the underlying data.	●	○	○	○	
M12. Need for extrapolation and/or need to integrate multiple data sources are considered.	●	○	○	○	
M13. Model validation - including an assessment of the model structure, assumptions, data, and results - is conducted.	●	○	○	○	
R10. Type of model used is clearly stated.	●	○	○	○	
R11. Justification of modeling choices and assumptions are provided. R12.	●	○	○	○	
Model descriptions are detailed enough to allow for replication. R13.	○	●	○	○	
Description of how model was validated is provided.	○	●	○	○	
	●	○	○	○	

© 2024 Tatara E et al. *JAMA Network Open*

Data Inputs and Evidence Synthesis

	Please select your level of agreement for each attribute.				Comments
	Yes	Somewhat	No	Not Applicable	
M14. A "best available evidence" approach is used to select data sources for model parameters (e.g., conducted or references systematic reviews/meta-analysis).	●	○	○	○	
M15. Data inputs are generated by appropriate statistical and epidemiological techniques.	●	○	○	○	
M16. Quality of the data (e.g. sources of bias) are assessed appropriately.	●	○	○	○	
R15. All data sources are referenced.	●	○	○	○	

© 2024 Tatara E et al. *JAMA Network Open*

Consequences

	Yes	Somewhat	No	Not Applicable	Comments
M17. Major consequences affected by the choice of interventions being compared are identified.	●	○	○	○	
R16. Comprehensive identification of consequences are summarized (e.g., using an Impact Inventory table in 2nd Panel's report).	●	○	○	○	

Please select your level of agreement for each attribute.

Utilities (Preference Measures)

	Yes	Somewhat	No	Not Applicable	Comments
M18. Health preferences reflect those of the jurisdiction(s) of interest (as specified in the decision problem).	●	○	○	○	
R17. Sources for the utility weights are clearly stated.	●	○	○	○	

Please select your level of agreement for each attribute.

Costs and Resource Use

	Yes	Somewhat	No	Not Applicable	Comments
M19. Resource use that is non-trivial in magnitude are included in the Reference Case analysis.	○	○	○	●	
R18. Quantities of resources are reported separately from the prices (unit costs) of those resources.	○	○	○	●	

Please select your level of agreement for each attribute.

© 2024 Tatara E et al. *JAMA Network Open*

Analysis

Analysis	Yes	Somewhat	No	Not Applicable	Comments
M20. Incremental analyses are conducted (i.e., the additional costs generated by one alternative over another are compared to the additional effects generated).	○	●	○	○	
M21. ICERs are obtained by comparing each intervention to the next most effective option, after eliminating dominated options.	○	●	○	○	
M22. Probabilistic sensitivity analysis is conducted to account for uncertainty in input parameters simultaneously.	●	○	○	○	
M23. Alternative modeling choices and assumptions (structural uncertainty) are explored through additional sensitivity analysis (i.e., scenario analysis).	●	○	○	○	
R19. Approach to secondary analyses (e.g., sensitivity, scenario, or subgroup analysis) is reported.	●	○	○	○	

Please select your level of agreement for each attribute.

Equity Considerations

Equity Considerations	Yes	Somewhat	No	Not Applicable	Comments
M24. Relevant equity or distributional considerations are taken into account.	●	○	○	○	
R20. Discussion section includes a description of any significant ethical implications of the CEA results.	●	○	○	○	

Please select your level of agreement for each attribute.

Transparency and Reporting

Transparency and Reporting	Yes	Somewhat	No	Not Applicable	Comments
R21. Results are presented in a disaggregated format for transparency.	●	○	○	○	
R22. Relevance of study results to specific decision problems are discussed.	●	○	○	○	
R23. Implications of uncertainty for decision-making, including the need for future research, are explored.	●	○	○	○	
R24. Potential bias and limitations are discussed.	●	○	○	○	

Please select your level of agreement for each attribute.

© 2024 Tatara E et al. *JAMA Network Open*

Final Score (Method 1): -

Methodological Quality: **89.5** out of 100 **(89.5%)**

Reporting Quality: **85** out of 100 **(85%)**

Final Score (Method 2): -

Methodological Quality: **85.5** out of **96** **(89.1%)**

Reporting Quality: **81** out of **96** **(84.4%)**

Cost-Effectiveness Analysis study evaluated: **An Agent-based model of combined community and jail-based take-home naloxone distribution**

Thank you for using the Criteria for Health Economic Quality Evaluation (CHEQUE) Tool.

If you have any questions or comments, please contact ddk@uchicago.edu

Powered by Qualtrics A

© 2024 Tatara E et al. *JAMA Network Open*

eAppendix 2. Justice-Community Circulation Model

The Justice-Community Circulation Model (JCCM) framework is an agent-based model (ABM) of opioid use (OU) in criminal legal system-involved (CLI) individuals who exit carceral settings. ABMs offer an ability to capture the nuances and complexities within public health crises and policies by combining a heterogeneous population of individual agent behaviors and detailed multi-level data (1,2). The JCCM framework incorporates information from detailed location-specific data sources on population demographics, drug use patterns and risk behaviors in the opioid-treatment cascade of care. JCCM includes a synthetic population of individuals who use illicit opioids, individuals with criminal-legal system involvement, and people who do not use drugs. We then incorporate variables that affect opioid-related risks and behaviors into our modeled demographic data. We incorporate these variables with specific attention to the variance of risk factors adjusted to demographic variables.

CLI and non-CLI individuals who use and are at-risk of using illicit opioids are modeled as autonomous agents who reflect the demographic and behavioral heterogeneity of the population. Each person agent possesses a set of unique demographic variables (e.g., age, sex, race) along with a set of time-varying behavioral variables (e.g., drug use and criminal legal system involvement). Demographic and behavioral variables are then combined with risk effects variables, which together determine the individual's probability of experiencing and surviving an opioid-related overdose (OOD).

Illicit drug use patterns exhibit dramatic regional and temporal variation. In Cook County IL, on which the current work is based, injection (3,4) and snorting (insufflation) (5) are the predominant routes of administration of non-prescription opioids. Although prescription opioid misuse does occur, declining rates of prescribing opioids (6) and the small proportion of overdose deaths involving prescription opioids in recent years (7) suggests that the driving factors in recent opioid overdose trends in Cook County, IL are due to illicit use of non-prescription opioids. Historically, opioid use has primarily involved heroin, but has recently included fentanyl and fentanyl-analogs in growing proportions (8). All of these forms are thus included in opioid use behaviors modeled in the JCCM.

© 2024 Tatara E et al. *JAMA Network Open*

eAppendix 3. Model Opioid Use Population Estimates

The JCCM uses a synthetic model population of approximately 140,000 unique individuals generated using demographic information collected from multiple Chicago-area studies and Chicago Department of Public Health data sets. Each of the model's opioid-use states represents individual agent states determined by the model population demographic data. We used a combination of several data sources to estimate the total number of people who misuse illicit opioids within Cook County, IL. The total population size of Cook County is approximately 5.2 million people in years 2015-2021, while the Illinois state total population is approximately 12.7 million during the same period. The City of Chicago population is approximately 2.7 million people during 2015-2021. Although the JCCM does not model the entire county population, we use the total population values to convert reported incidence rates per total population.

Estimating the locale-specific number of individuals who misuse opioids is challenging, because the task requires combining information from multiple data sources that originate from drug use self-reporting, from publicly available hospital discharge data statistics, and from opioid-related overdose deaths reported from Medical Examiners' and Coroners' offices. The State of Illinois reported a total of 1,382 opioid-related overdoses deaths in 2015 and has used the approach, attributed to the CDC, of estimating a ratio of 130 individuals living with OUD for every reported opioid overdose death to estimate the state total number of individuals with OUD at approximate 180,000 (9). Cook County accounts for nearly 50% of the State's opioid related overdose deaths in 2015-2016 (9). If we assume that the fraction of opioid overdose deaths in Cook County relative to the State reflects the size of the population of individuals living with OUD, then we estimate that the total Cook County opioid use (OU) population at approximately 90,000 individuals. A recent report by Luo (10) estimates the Illinois state OUD population in 2017 at approximately 70,000 individuals using National Survey on Drug Use and Health (NSDUH) 2-Year Restricted-Use Data Analysis System (2016–2017). The JCCM OU population includes individuals who are simply at risk of opioid-related overdose, and not specifically individuals who have been clinically diagnosed as having OUD.

Estimating the opioid use population as a function of reported overdose deaths is particularly challenging for recent years, given the significant rise in opioid-related overdose deaths, and specifically deaths involving fentanyl. In 2020, the State of Illinois reported 2,944 opioid-related overdose deaths (11), representing a more than twofold increase in opioid overdose deaths compared to 2015. Using the same CDC estimate for OUD population as 130x the number of reported opioid overdose deaths would result in an OUD population size for Illinois of 383,000 individuals in 2020 - double the size of the OUD population in 2015. While the rise in opioid overdose deaths may be due to a combination of an increase in the OUD population size, social factors, and increased lethality of illicit opioids, the JCCM model assumes a constant population size of 90,000 Cook County residents who use heroin, fentanyl, and other opioids based on these 2015 estimates.

Within JCCM, the population of people who use opioids is then divided into two key subpopulations: non-injection opioid users (NIOU), and injection opioid users (IOU). For

© 2024 Tatara E et al. *JAMA Network Open*

simplicity, JCCM considers individuals to be primarily either injectors or non-injectors in distinct subpopulations.

A population size of 24,000 IOU in Cook Co. is estimated using published population models (12) and demographics (3) of persons who inject drugs (PWID). Our PWID population estimates are based on data published in previous simulation studies (13,14) that estimates the PWID population of the metropolitan Chicago area (15) at 32,000 individuals using PWID estimates from Tempalski et al (12) for the Chicago metropolitan statistical area that includes the a multi-county Chicago-Gary-Kenosha, IL-IN-WI region. Details on the generation of the synthetic population was previously described (14). In brief, parameter estimates were generated to profile each of the estimated 32,000 PWID (16) residing in metropolitan Chicago represented in the synthetic population [CNEP+] from analyses of two empirical datasets. These were the (i) 2009 metropolitan Chicago PWID data from the CDC-sponsored National HIV Behavioral Surveillance survey (17) of 545 PWID [NHBS 2009] and (ii) 2006-2013 data from large, multi-site syringe service program (SSP) of >6,000 participants (15) [CNEP]. Of the total 32,000 PWID sampled from the CNEP+ dataset, approximately 24,000 of the individuals were located within Cook Co.

The size of the NIOU subpopulation size in Cook Co. is estimated simply by subtracting the size of the estimated IOU population (24,000) from the size of the estimated opioid use population (90,000) to arrive at a subpopulation of 66,000 individuals. The non-injecting subpopulation demographics are estimated using those published for non-injection heroin users (NIHU) in Chicago (5) and other locales (18). The estimated fractions of injecting and non-injecting sub-populations used in the JCCM is in line with previous reports of individuals who were enrolled in publicly funded treatment programs in Chicago in 2012 and their reporting non-injection vs injection as the primary route of administration (19).

© 2024 Tatara E et al. *JAMA Network Open*

eAppendix 4. Cook County Jail Population Estimates

The nightly census of the Cook County jail varied considerably, with a peak population of more than 10,000 individuals in 2002-2003 (20), and has declined to a population size of 5,500 – 6,500 in years 2018-2021 (20–22). JCCM uses a daily average jail population of 6,000 individuals which is consistent with the Cook County jail population in recent years. Recent published data on Cook County jail population dynamics (2011) reflect an average of 196 admissions per day with a total of 71,663 annual admissions and an average daily population of 8,896 (20). Using a model average daily population of 6,000, we estimate a daily average of 132 admissions using the same ratio of daily admissions to average daily population for 2011. To maintain an approximately constant average daily jail population, the number of daily jail discharges is kept equal to the number of daily admissions.

Estimates for length of incarceration (LOI) are also available, with a mean of 54.1 days spent in jail, and median of 12 days. One-quarter of discharges are associated with jail stays of two or fewer days. with 3% spending more than one year in jail (20). The same 2011 admissions data show that 83.2% of individuals were admitted only once, while 13.4% of individuals had two admissions during the year, and 3.4% of individuals had three or more admissions (20).

The jail circulation model in JCCM considers prisoner admission and release to and from a constant population of criminal legal system-involved (CLI) individuals in Cook Co. Although nearly 20% of all discharges were transfers to prison in 2011 – 2012 (20,23) with the remaining 80% discharges being some form of release, JCCM does not directly or separately model the prison population. It assumes all prisoner discharges are released. JCCM models a CLI population size of 50,000 individuals. Individual-level demographic details for all prisoners of the Cook County jail system are obtained from the Cook County Sheriff's website years 2016-2017 (24). Although the total jail population size has changed significantly in the past decade, the demographic distribution of inmates has not, thus providing an opportunity to leverage the fine-grained demographic details from years 2016-2017 when modeling future years

JCCM models LOI by sampling from an empirically fit distribution to determine the LOI for every individual that is booked into jail. The LOI distribution is defined by the piecewise distribution as follows:

- 25% of all booked persons are assigned a LOI of between 1-2 days
- Another 25% of all booked persons are assigned a LOI of between 3-12 days
- The remaining 50% of all booked persons are assigned an LOI sampled from a Weibull distribution between 14 and approximately 1,000 days.

A random sampling of 50,000 persons produces a LOI distribution that closely matches the above referenced statistics for the Cook County Jail and the summary statistics are provided below. The modeled LOI distribution does not account for demographics or seriousness of the crime for which the persons was arrested.

© 2024 Tatara E et al. *JAMA Network Open*

eTable 1. Summary Statistics for the JCCM Generated Length of Incarceration Distribution (Days)

Statistic	Length of Incarceration (days)
**Min**	1
**Max**	923
**Median**	12
**Mean**	53.28
**25th Percentile**	2
**50th Percentile**	12
**90th Percentile**	157
**95th Percentile**	220
**99th Percentile**	370

© 2024 Tatara E et al. *JAMA Network Open*

eAppendix 5. Opioid Use Among Arrestees

Recent data are sparse regarding opioid use within the Cook Co. jail. Local media provide some coverage of ongoing opioid treatment programs in the jail, indicating a monthly enrollment in medication-assisted treatment (MAT) programs at 375-400 individuals (25). Considering the 2019 Cook Co. jail population size of 5,500 – 6,000, a conservative estimate of the percentage of jail inmates with current opioid use at 6 – 7%. The number of individuals enrolled in MAT programs is likely significantly fewer than the number of incarcerated individuals who are opioid users.

The Arrestee Drug Abuse Monitoring II (ADAM II) survey provides data on drug use from booked male arrestees in select US counties in years 2008-2013, including Cook County (26). The ADAM II samples for Cook County provide data that can help estimate the fraction of arrestees testing positive for opioids, but this sample is not representative of the jail population because it was collected only in the main Cook Co. Jail facility for male arrestees with felony and serious misdemeanant charges. The most recent available data (from 2013) indicate that 14.3% (std error 3.0%) of male arrestees have urine tests positive for opiates, 11.8% report using heroin in the past 12 months (26), and 15.5% (3.3% s.e.) report acquiring heroin in the last 30 days, with an average of 19.1 (2.4 s.e.) heroin purchases in the last 30 days and an average number of 21.2 (2.5 s.e.) of days using heroin in the past 30 days used. Of those who did report heroin use, 26.2% (10.7 s.e.) reported injection at the most recent use.

Demographics for arrestees testing positive for opiates in 2013 also include:

- Percent positive for opiates by age:
  - <21: 2.2%
  - 21-25: 7.8%
  - 26-30: 16.6%
  - 31-35: 10.5%
  - 36+: 20.6%
- Percent positive for opiates by race:
  - White: 22.8%
  - Black: 10.0%
  - Hispanic: 10.7%
  - Other: 7.9%

We estimate the percent of the CLI individuals who are opioid users for contemporary Cook Co. by using the ADAM II 2013 values of 14.3% of arrestees testing positive for opiates, with a 95% CI of +/- 6%, representing a lower estimate of approximately 8% and upper estimate of approximately 20%. The percent of ADAM II arrestees (26.2%,) who report injection at most recent heroin use is used to estimate the fraction of jail inmates who are primary injectors and non-injectors.

© 2024 Tatara E et al. *JAMA Network Open*

eTable 2. JCCM Input Values for the Model Population Sizes

Parameter	Nominal Value	Lower Estimate	Upper Estimate
**Total OU Population size**	90,000	N/A	N/A
**IOU population size**	24,000	N/A	N/A
**NIOU population size**	66,000	N/A	N/A
**CLI population size**	50,000	N/A	N/A
**Jail average daily population**	6,000	N/A	N/A
**Percent of jail inmates who use any opioids**	14.3%	8%	20%
**Percent of jail inmates who use opioids primarily through injection**	3.75%	2%	5%
**Percent of jail inmates who use opioids primarily through non-injection**	10.55%	6%	15%

eAppendix 6. JCCM Synthetic Population Generation

For each model run, the synthetic model population of individuals is generated using the population size parameters from §3.3. First, the total model population of opioid users is generated, resulting in an OU population whose demographics matches the demographic data for NIOU and IOU persons in Cook County described above. Next, the CLI population is generated using the demographics from the Cook Co Jail described above. The model CLI population includes persons currently in jail, and persons who may be arrested during the simulation timeframe. Persons in the CLI population who are opioid users are iteratively sampled from the total OU population such that both the percent of inmates who whose opioids match the model input values in §3.3 and the CLI population demographics match the values reported above for Cook County Jail.

The data below show a sample demographics for the non-CLI OU for a single model run using the population totals in §3.3. When the model is initialized, each demographic value is used to generate the corresponding number of individuals in the model. For example, using the data below, the model will generate 1,249 individuals who are black females ages 18-24 NIOU, and 611 non-Hispanic white females ages 18-24 NIOU, etc. The non-CLI population includes only individuals who are opioid users. The JCCM does not model non-CLI individuals without a history of opioid use.

The data for the total CLI population of 50,000 individuals is generated using the demographics for Cook County Jail, and values for percent of inmates who are primarily noninjecting and primarily injection opioid users of 10% and 3.5%, respectively. The CLI population includes non-opioid users, and this demographic constitutes the majority of the CLI population.

© 2024 Tatara E et al. *JAMA Network Open*

eTable 3. A Sample Generated JCCM Non-CLI Population Showing the Numbers of Individuals for Non-Injection Opioid Users (NIOU) and Injection Opioid Users (IOU)

	Sex	Female		Male	
	OU State	IOU	NIOU	IOU	NIOU
Race	Age				
Black	18-24	154	1249	136	2900
	25-34	127	3737	124	8678
	35-44	529	3579	601	8312
	45+	970	1278	1922	2969
Hispanic	18-24	139	664	262	1541
	25-34	449	1985	940	4611
	35-44	426	1902	1036	4417
	45+	282	679	1194	1577
NHWhite	18-24	994	611	1861	1418
	25-34	2355	1827	4892	4242
	35-44	737	1750	1782	4064
	45+	263	625	1108	1451
Other	18-24	40	0	84	0
	25-34	92	0	214	0
	35-44	38	0	104	0
	45+	26	0	122	0

© 2024 Tatara E et al. *JAMA Network Open*

eTable 4. A Sample Generated JCCM CLI Population of 50,000 Individuals Showing the Numbers of Individuals for Non-Injection Opioid Users (NIOU) and Injection Opioid Users (IOU), Assuming a Fraction of CLI Individuals Who Are IOU of 3.5% and a Fraction of CLI Individuals Who Are NIOU of 10%

	Sex	Female			Male		
	OU State	IOU	NIOU	Non OU	IOU	NIOU	Non OU
**Race**	**Age**						
**Black**	**18-24**	11	95	637	10	220	11821
	**25-34**	9	283	548	9	657	9173
	**35-44**	39	271	250	44	630	4591
	**45+**	71	97	644	140	225	6061
**Hispanic**	**18-24**	10	50	152	19	117	2345
	**25-34**	33	150	51	69	349	2046
	**35-44**	31	83	0	76	335	1173
	**45+**	21	30	0	87	119	874
**NHWhite**	**18-24**	72	19	0	136	107	632
	**25-34**	172	138	27	357	321	671
	**35-44**	54	133	13	130	308	511
	**45+**	19	47	60	81	110	987
**Other**	**18-24**	0	0	0	6	0	0
	**25-34**	6	0	0	16	0	7
	**35-44**	0	0	0	6	0	0
	**45+**	0	0	0	9	0	20

© 2024 Tatara E et al. *JAMA Network Open*

eAppendix 7. Estimating Opioid-Related Overdose and Deaths

Opioid-related overdose deaths are reported by the Cook County Medical Examiner for every detected case of opioid poisoning for years 2014-2022 (27). These Medical Examiner data are used to generate cross tabulations for all demographics such as age, race, and sex for the purpose of defining model calibration targets. Counts of annual opioid poisoning deaths by demographic category are used to estimate probabilities of fatal and non-fatal overdose, and are used in validation of model predictions of yearly overdose deaths.

Estimating total yearly non-fatal opioid overdoses at the county and state levels requires examination of sources that report hospital emergency department (ED) discharge data and emergency medical services (EMS) calls for opioid overdoses. The number of non-fatal opioid overdoses per year are used to estimate the probability of overdose for the population of individuals who are opioid users and the fraction of all opioid overdoses that are fatal. The IDPH Opioid Data Dashboard (28) provides data on non-fatal opioid overdoses by examining hospital discharge data for inpatient and outpatient patient discharges with ED department billing and ICD-9/10 codes for poisoning with opioids. The yearly counts of opioid-related patient discharges for Cook Co. are shown in. The IDPH reported hospital discharge data for non-fatal opioid overdoses likely represents a conservative lower estimate on the yearly rates as this includes only cases of overdose in which the individual was diagnosed and treated by a physician. Individuals who overdose and do not seek medical attention would not be included in this count of overdose cases.

The Chicago Department of Public Health (CDPH) provides counts of EMS calls per year for suspected opioid overdoses and includes calls reported by Chicago Fire Department (CFD) EMS for suspected opioid overdoses when naloxone has been administered (29,30). The Chicago yearly EMS calls are larger in number than the number of reported non-fatal overdoses for all of Cook County as reported by IDPH which suggests that using the number of hospital discharges for opioid poisoning significantly underestimates the actual number of yearly opioid overdose cases. Considering that the population size of Chicago is roughly 50% of Cook Co., and comparing the number of yearly overdose cases in Cook Co to those occurring in the City of Chicago, we estimate an upper bound on the total yearly number of non-fatal overdoses in Cook Co. as twice those reported by CFD EMS. The CDPH reports of Chicago EMS calls does not indicate the survival rate of overdoses. However if these cases reflect naloxone administration, then the survival rate is over 95%.

The Healthcare Cost and Utilization Project (HCUP) provides estimates for “opioid-related” ED visits and inpatient stays at the national and state level, but include ICD-9/10 codes for opioid dependence and abuse in addition to diagnosis codes for poisoning by opioids (31,32) which likely is a significant over-estimate of the rates of opioid poisoning. For a large central metro location in Illinois, the rates of opioid-related inpatient stays for 2014 are estimated at 518/100,000 population, and the rates of opioid-related ED visits are estimated at 235 / 100,000 population (33). These rates would reflect a total of 37,650 opioid-related inpatient stays and ED visits for Cook Co. in 2014. Because the HCUP data likely over-estimate opioid poisoning rates,

© 2024 Tatara E et al. *JAMA Network Open*

these rates are provided merely for reference and are not used to inform the estimates of overdose used in the JCCM.

© 2024 Tatara E et al. *JAMA Network Open*

eTable 5. Yearly Opioid-Related Overdose Deaths (OODs) in Illinois State and Cook County, IL

Year	2015	2016	2017	2018	2019	2020	2021	2022
Illinois OODs (11,28,34)	1,382	1,946	2,202	2,167	2,219	2,944	3,013	3,261
Cook Co. OODs (IDPH) (28)	599	1,236	1,446	1,455	1,622	2,278	2,179	2,128
Percent of Cook Co. Deaths / IL Deaths	43%	64%	66%	67%	73%	77%	72%	65%
Chicago OODs (35)	426	741	796	796	855	1,303		
• Heroin Involved	345	487	575	520	501	638		
• Fentanyl Involved	NA	420	470	620	683	1,123		
• Fentanyl Only			145	212	288	500		
Cook Co. OODs (Medical Examiner) (36–38)	676	1,126	1,195	1,170	1,295	1,846	1,938	2,001
• Heroin Involved		693	787	680	666	778	608	412
• Fentanyl Involved		560	669	847	961	1533	1721	1842
• Fentanyl Only								
• Rx Opioid Involved		177	211	197	188			

Notes:

- Cook County Medical Examiner reports deaths that occur in Cook County with opioid overdose as primary cause.
- The IDPH data dashboard reports deaths from IDPH Division of Vital Records for overdose deaths of individuals whose residence address is Cook County.
- Cook County Medical Examiner began testing for fentanyl in mid-2015.

© 2024 Tatara E et al. *JAMA Network Open*

eTable 6. Data Used to Estimate Yearly Non-Fatal Opioid Overdose Events in Cook County, IL

Year	2015	2016	2017	2018	2019	2020	2021	2022
Cook Co. non-fatal overdoses (28)	3,080	5,548	6,962	7,585	8,852	12,205	11,328	10,429
Cook Co. EMS Events (IDPH)	3,500-4,500 (9)							
Cook Co. ED Visits (IDPH)	500-1,750 (9)	2,500-3,500 (39)						
Chicago EMS Calls	2,978 (29)	6,590 (29)	7,526 (29)	8,159 (29)	10,490 (29)	13,794 (35)		

Notes:

- Data are unavailable for blank cells
- IDPH non-fatal overdoses from IDPH hospital discharge
- EMS calls reported by Chicago Fire Department EMS for suspected opioid overdoses when naloxone has been administered

© 2024 Tatara E et al. *JAMA Network Open*

eTable 7. Estimated Ranges of Yearly Fatal and Non-Fatal Opioid Overdoses for Cook Co., IL

Year	2015	2016	2017	2018	2019	2020	2021	2022
Non-fatal Overdoses lower bound	3,080	5,548	6,962	7,585	8,852	12,205	11,328	10,429
Non-fatal Overdoses upper bound	5,956	13,180	15,052	16,318	20,980	27,588		
Fatal Overdoses	676	1,126	1,195	1,170	1,295	1,846	1,938	2,001
All Overdoses lower bound	3,756	6,674	8,157	8,755	10,147	14,051	13,266	12,430
All Overdoses upper bound	6,632	14,306	16,247	17,488	22,275	29,434		

- Data are unavailable for blank cells
- Non-fatal Overdoses lower bound is estimated as the number of hospital discharges in Cook Co reported by IDPH
- Non-fatal Overdoses upper bound is estimated as twice the number of Chicago Fire EMS responses for suspected opioid-related overdoses

For every year t, we estimate the annual probability that an individual who is an opioid user will experience an opioid overdose as the ratio of the number of all overdose events in the year divided by the opioid use population:

$$P(OD,t) = \frac{number\ of\ fatal\ overdoses + number\ of\ nonfatal\ overdoses}{size\ of\ the\ opioid\ use\ population}$$

The probability that an overdose event will be fatal is the ratio of the number of fatal overdoses to the number of all overdoses in the year:

$$P(fatal,t|OD) = \frac{number\ of\ fatal\ overdoses}{number\ of\ fatal\ overdoses + number\ of\ nonfatal\ overdoses}$$

Thus, the annual probability of an individual experiencing a fatal overdose is:

$$P(fatal\ OD,t) = P(OD,t) \times P(fatal,t|OD)$$

The annual probability of a fatal or non-fatal overdose event can be converted to a weekly or daily probability by dividing the annual probability by 50 or 365, respectively.

JCCM input values for overdose rates and probabilities are shown for year 2018 and 2020 are shown below, including lower and upper estimates for non-fatal overdoses. The model uses a

daily time step of 1 day and therefore overdose probabilities are input as daily probabilities. Nominal values are the mean of the lower and upper estimates.

© 2024 Tatara E et al. *JAMA Network Open*

eTable 8. JCCM Input Values for Overdose Rates and Probabilities for Simulation Year 2018

Parameter	Nominal Value	Lower Estimate	Upper Estimate
**Fatal overdoses**	1,170	N/A	N/A
**Non-fatal overdoses**	12,000	7,585	16,318
**Total overdoses**	13,146	8,755	17,464
**Daily overdose probability per person**	0.040%	0.027%	0.053%
**Fatal probability per overdose**	8.7%	6.6%	13.1%

© 2024 Tatara E et al. *JAMA Network Open*

eTable 9. JCCM Input Values for Overdose Rates and Probabilities for Simulation Year 2020

Parameter	Nominal Value	Lower Estimate	Upper Estimate
**Fatal overdoses**	1,846	N/A	N/A
**Non-fatal overdoses**	19,897	12,205	27,588
**Total overdoses**	21,743	14,051	29,434
**Daily overdose probability per person**	0.066%	0.043%	0.089%
**Fatal probability per overdose**	8.5%	6.3%	13.1%

© 2024 Tatara E et al. *JAMA Network Open*

eTable 10. Nominal Non-Fatal and Fatal Overdose Probabilities per Year in Cook Co. IL

Year	2015	2016	2017	2018	2019	2020
Daily overdose probability per person	0.016%	0.032%	0.037%	0.040%	0.049%	0.066%
Fatal probability per overdose	12.5%	10.5%	9.6%	8.7%	7.8%	8.5%

© 2024 Tatara E et al. *JAMA Network Open*

eAppendix 8. Variables That Affect Overdose Risks and Survivals

The probabilities of non-fatal and fatal overdose described in the prior section are general to the entire population of people who use opioids in Cook Co. Demographic and behavioral risk factors are included in JCCM to modify a modeled individual's probabilities of overdose and death from overdose based on published risks from the literature.

An individual's risk-adjusted probability of overdose is defined as the base overdose probability multiplied by the total product of every associated overdose risk x_i for that individual:

$$P'(OD,t) = P(OD,t) \times \prod x_i$$

Overdose Risk Parameters
Risk factors for opioid overdose are generally reported for fatal overdoses, as univariate or adjusted hazard ratios, risk ratios, and odds ratios. JCCM considers these hazards as affecting the probability of any overdose because it is not possible to decouple risks for fatal and non-fatal overdoses based on the published data.

The model parameter data below for overdose risk is informed by published opioid overdose risks and generally reflect published values for hazard ratios, however we also use published odds ratios and SMR to inform the selection of nominal and bounded estimates for these risk parameters.

Jail post-release overdose risk
There are limited published studies that report consistent increased risk of fatal and nonfatal overdose among recently released prison inmates (40–42), and a single study that reports the increased risk among those recently released from NYC jail (43). As the reported rate of overdoses following jail release are lower than the rates following prison release, the range of values for this risk parameter as applied to a study on the Cook County jail is selected carefully based on expert opinion to adjust this risk to account for recent trends in overdoses and the effects of fentanyl availability in Cook County.

© 2024 Tatara E et al. *JAMA Network Open*

eTable 11. Model Risk Factors That Affect Overdose Probability

Parameter	Nominal Value (relative risk compared with reference)	Lower Estimate	Upper Estimate	Citations
**Male**	1.0 (reference)			(42,44,45)
**Female**	0.62	0.21	1.41	(42,44,45)
**Non-injection user**	1.0 (reference)			(45)
**Injection user**	2.1	0.5	9.1	(45)
**Jail release week 0-2**	6	2	10	(40,46–48)
**Jail release week 3-4**	4	2	6	(40,46–48)
**Jail release week 5+**	2	1	2	(40,46–48)
**Age < 25**	1.0 (reference)			(45)
**Age 25-29**	1.22	0.39	3.78	(42,45,47)
**Age 30+**	1.92	0.72	5.15	(45,47)

© 2024 Tatara E et al. *JAMA Network Open*

eAppendix 9. Naloxone Intervention Variables

Naloxone distribution and take-home naloxone effectively lowers opioid overdose mortality by up to 95% (49,50). Take-home naloxone is available in a nasal spray that is typically administered by a bystander such as a family member, peer drug-user, or other social acquaintance, as the person experiencing the overdose will usually be unconscious. Naloxone availability to the public is increasing, primarily though the equipping of EMS responders with kits, and through distribution via drug treatment and harm reduction programs. The model assumes that the probability of OD reversal with naloxone is independent of dosage, although it is common for THN kits to contain at least two doses. The model further assumes that naloxone wastage and loss does not occur, and that individuals immediately obtain a new THN kit after using an existing kit to reverse an overdose (see assumptions and limitations section).

The modeled intervention of naloxone distribution in JCCM includes three separate, but complementary, avenues of distribution:

1. Inmates who are opioid users may be provided with multi-dose take home naloxone (THN) kits upon release from jail. The percentage of inmates provided with THN kits upon release is a model parameter in the sensitivity analysis. Released inmates who receive THN may have an overdose reversed if a bystander is present.

2. Naloxone may be distributed within the opioid use and CLI community which provides an opportunity for community-distributed naloxone to be used by a bystander who witnesses an overdose event. The amount of community-distributed naloxone is a model parameter in the sensitivity analysis.

3. Targeted naloxone distribution to the social network of released jail inmates. The percent of the released individuals' social network who have possession of THN is a model parameter in the sensitivity analysis.

© 2024 Tatara E et al. *JAMA Network Open*

eAppendix 10. Model Agent Behavior Logic

The JCCM uses a time step of 1 day to determine opioid-related overdose events and assumes that each individual's yearly overdose probability is distributed equally over the year, with daily probability of overdoses set equal to the yearly probability / 365. Thus, the model explicitly simulates daily drug use behaviors for individuals who are opioid users, but the overdose risk can be aggregated over any time-period (day, week, month, or year). Opioid users who are currently incarcerated are presumed not to engage in daily drug use activities until they are released from jail.

For every individual on each day:

1. Calculate the probability of overdose as a function of the base overdose probability and associated hazards specific to the individual, $P'(OD, t)$, described above.

2. Determine if an overdose event occurs by drawing a random uniform variable *a* ~ [0,1] and IF *a* < P(OD), an overdose event has occurred.

3. IF an overdose (OD) event has occurred, determine if the overdose is fatal or non-fatal. This probability is dependent on the following random variables for the probability of OD survival:
   - The probability of survival when naloxone not administered:
   $$P(survival) = 1 - P(fatal\ OD, t \mid OD)$$
   - The probability of survival when naloxone is administered, $P(survival \mid N)$
   - The probability of death is either:
   $$P(death) = 1 - P(survival), \text{ or}$$
   $$P(death) = 1 - P(survival \mid N)$$

Naloxone administration to reverse an overdose requires a bystander in the presence of the person experiencing the overdose. The probability that a bystander is present is a random variable described above.

The joint probability of surviving an overdose <u>when a bystander is present</u>, and naloxone is available is:

- P(naloxone available) x P(naloxone effectively administered) x P(survival | N)
- P(survival | N) is a constant model parameter that reflects the reported probability of reversing an overdose with naloxone, which is reported in the literature at > 95%.

© 2024 Tatara E et al. *JAMA Network Open*

- P(naloxone available) is the probability that the individual or bystander was provided with naloxone.
- P(naloxone effectively administered) is a model parameter for sensitivity analysis in the range 20% to 90%. This parameter lumps the probability that the provided naloxone was immediately accessible (present at the overdose location) and administered correctly and promptly.

When a bystander is not present at an overdose, the probability of survival is:

- The probability of survival when naloxone not administered, P(survival)

The probability that naloxone will be available during an OD event, P(naloxone available), is a function of the probability that:

- the individual experiencing the OD was directly provided naloxone. We simplify this to be:
    - P() = 1.0 if the individual was provided a THN kit at release from jail.
    - P() = 0, if the individual was not provided a THN kit at release from jail.
- a bystander is present during the OD and was provided with naloxone

The probability that a bystander was provided with naloxone is a function of:

- the level of community distribution of THN, or
- the level of social network distribution of THN

and can be defined as the combination of these two avenues of THN distribution.

© 2024 Tatara E et al. *JAMA Network Open*

eAppendix 11. Model Software Implementation and Model Parameter Screening

We used the Repast4Py (51) distributed ABM toolkit to implement the JCCM. Repast4Py is part of the open-source Repast ABM Suite of toolkits which in addition to Repast4Py consists of Repast Simphony and Repast for High Performance Computing (RepastHPC) (52). Repast4Py leverages our experience in developing and using RepastHPC, also a distributed ABM toolkit targeted at HPC systems, while swapping Python for C++ as the ABM implementation language. Python has become the primary programming language for artificial intelligence (AI) and is widely used across many scientific disciplines for machine learning (ML) and other applications. It also boasts a vibrant ecosystem of libraries that exploit the latest advances in algorithmic development and emerging hardware architectures.

We used the EMEWS framework (53) and the underlying Swift/T workflow engine (54) to implement the JCCM sensitivity analysis as an HPC workflow. EMEWS provides the ability to define resident, or stateful, tasks written in programming languages such as R, Python, and Julia, to encapsulate the logic of iterating, state-preserving algorithms. These resident tasks directly control the logical flow of the overall workflow through high-level queue-like interfaces. Thus, complex algorithms can be applied at cluster and leadership computing scales. We also utilized new capabilities in Swift/T for launching distributed mpi4py Python applications via the *python_persist_parallel* call, which passes an MPI sub-communicator to an embedded Python interpreter. This allows for efficient launching and coordination of large numbers of Python MPI applications.

Model Parameter Screening

Model input parameter screening was performed using the Sobol method to estimate the relative contributions of each model input parameter to the model response variables outputs [38]. Sobol total effects indices for each input parameter provide a quantitative measure (percent contribution) of both first order and parameter interaction effects on the total variance explained by the model outputs. The size of the parameter space for the sensitivity analysis can be reduced by identifying only those model input parameters with the greatest effect on model output variance. Screening was performed using the R *sensitivity* package *soboljansen* (Jansen, 1999; Saltelli et al., 2010). A total of 47,500 individual JCCM simulation runs were executed on the Argonne LCRC bebop cluster using random sampling of the parameter space.

Non-intervention parameters are defined as those that represent contextual variation in the modeled population. Obtaining point estimates for the non-intervention parameters is challenging, and therefore a lower and upper bound on the parameter values was used to constrain the sensitivity analysis. The Sobol total indices for deaths averted for the entire model OU population, and deaths averted for the population of persons released from jail, ranked by number of deaths averted in the released persons group are shown below. These values represent the approximate proportion that each model input (row) contributes to observed

variability of the deaths averted predicted (columns) by the model. The sum of each column is approximately 1.0 but can exceed 1.0 due to interaction effects between input variables.

The probability of bystander presence at the location of overdose is the model parameter with the greatest proportional contribution (0.2715 or 27.15%) to variance in deaths averted in persons released from jail. Also particularly important are the base probability of overdose (0.2672 or 26.72%) and the intervention of naloxone distribution to persons released from jail (0.2135 or 21.35%).

An exhaustive sensitivity analysis of all model input parameters and their effects on model response variables is computationally infeasible. To reduce the number of model parameter combinations, we selected the three naloxone intervention parameters, and the top five non-intervention parameters of interest, from which to randomly sample between the parameter value bounds. Furthermore, to avoid sampling from an infinite number of continuous values between the parameter bounds, we categorized sensitivity analysis parameters into upper and lower parameter values bounds, and their midpoint.

For each of the 27 possible naloxone intervention scenarios, we varied the five non-intervention parameters across 243 combinations of high, medium, and low values, which provides sufficient variation in the model results to simulate the spread of expected real-world conditions. Ten stochastic replicates were executed for each of the 243 combinations of non-intervention parameters, resulting in 2,430 individual simulation runs for each of the 27 intervention scenarios, from which the statistics were computed. This approach produced a grand total of 65,610 individual simulation runs.

For each naloxone intervention scenario we report the mean, 25th, and 75th percentiles for the total number of naloxone kits distributed, percent (pct) of opioid-related deaths averted, cost per death averted, and number needed to treat (NNT) to avoid one opioid-related overdose death. Cost per death averted is the total intervention cost (community + jail + jail network) divided by the number of overdose deaths averted in the entire model OU population. NNT to prevent one opioid-related overdose death is defined as the number of naloxone kits distributed divided by the number of opioid-related overdose deaths averted.

A baseline scenario (Scenario 1) with no naloxone intervention present is used to compare the number of deaths averted in the other scenarios that do model active naloxone interventions. Scenarios 2 through 27 indicate varying levels of the three naloxone intervention parameters. The variance demonstrated by the 25th and 75th percentiles for each model response is due to the sampling of the non-intervention parameters in combination with the stochastic replicates.

© 2024 Tatara E et al. *JAMA Network Open*

eTable 12. Sobol *T*otal *S*ensitivity *I*ndices for *D*eaths *A*verted for the *E*ntire *M*odel OU *P*opulation, and *D*eaths *A*verted for the *P*opulation of *P*ersons *R*eleased *F*rom *J*ail, *R*anked by *N*umber of *D*eaths *A*verted in the *R*eleased *P*ersons *G*roup

The values below represent the approximate proportion (fraction) that each model input (row) contributes to observed variability in the deaths averted predicted (columns) by the model. The sum of each column is approximately 1.0, but can exceed 1.0 due to interaction effects between input variables.

**Model Inputs**	**Model Responses**	
	Deaths Averted in the entire OU population	Deaths Averted among people released from jail
**Probability of a bystander present during overdose**	0.2532	0.2715
**Daily overdose probability per person**	0.2165	0.2672
**Naloxone distribution to individuals released from jail**	0.0052	0.2135
**OD risk, Age 35-44**	0.0809	0.0984
**Percent of jail inmates non-injection (NIOU)**	0.0035	0.0974
**Probability of administering naloxone during overdose**	0.0686	0.0879
**OD risk, Age 25-34**	0.0585	0.0844
**Fatal probability per overdose**	0.0386	0.0696
**Probability of bystander calling EMS during overdose**	0.0342	0.0569
**OD risk, Female**	0.0363	0.0464
**OD risk, Jail release week 5+**	0.0015	0.0399
**OD risk, Age.45+**	0.0238	0.0381
**Naloxone distribution to the OU community**	0.3769	0.0381
**Naloxone to social network of individuals released from jail**	0.0014	0.0320
**Percent of jail inmates injection ()**	0.0017	0.0307
**OD risk, Jail release week 0-2**	0.0014	0.0303
**OD risk, Jail release week 3-4**	0.0010	0.0189

© 2024 Tatara E et al. *JAMA Network Open*

eTable 13. Non-Intervention Parameters Values for Intervention Scenarios Analysis

Parameter Name	Low Value	Medium Value	High Value
**Daily overdose base probability**	0.016%	0.033%	0.05%
**Fatal overdose base probability**	7%	10%	13%
**Probability of bystander present**	30%	60%	90%
**Probability that naloxone is administered**	50%	70%	90%
**Fraction of CLI population NIOU**	6%	10.5%	15%

© 2024 Tatara E et al. *JAMA Network Open*

eTable 14. Intervention Parameter Values for Intervention Scenarios Analysis

The percentage values represent the percent of individuals that receive naloxone.

Parameter Name	Low Value	Medium Value	High Value
**Jail naloxone distribution**	0%	50%	100%
**Jail network naloxone distribution**	0%	15%	30%
**Community naloxone distribution**	0%	15%	30%

© 2024 Tatara E et al. *JAMA Network Open*

eAppendix 12. Assumptions and Limitations of the JCCM

- We assume that incarcerated individuals are not actively using within the secure setting, although in reality some incarcerated individuals would have access to illicit drugs in jail.
- We do not consider prescription opioid misuse.
- Fentanyl presence in a dose of illicit opioids includes any fentanyl analog. Different forms of fentanyl have varying levels of risk of potency and risk of overdose. Our model lumps the probability of fentanyl-related overdose and death risk by using medical examiner data for all fentanyl analogs.
- The modeled LOI distribution does not account for demographics or seriousness of the crime for which the person was arrested.
- The model assumes that the probability of OD reversal with naloxone is independent of dosage, although it is common for THN kits to contain at least two doses.
- The model does not include naloxone wastage and loss
- The model assumes that individuals immediately obtain a new THN kit after using an existing kit to reverse an overdose.
- Formerly-incarcerated persons may only directly obtain THN upon release from the secure setting, and are assumed to obtain refills after use. This is independent of community level naloxone distribution. Released inmates who are not provided with naloxone at release do not have an opportunity to receive naloxone from the community.
- Community level THN distribution is only provided to individuals who are not recently released from incarceration.
- Social networks are not modelled explicitly. The model defines the probability of an individual with a social network connection who witnesses the overdose and is in possession of naloxone as a probability.

© 2024 Tatara E et al. *JAMA Network Open*

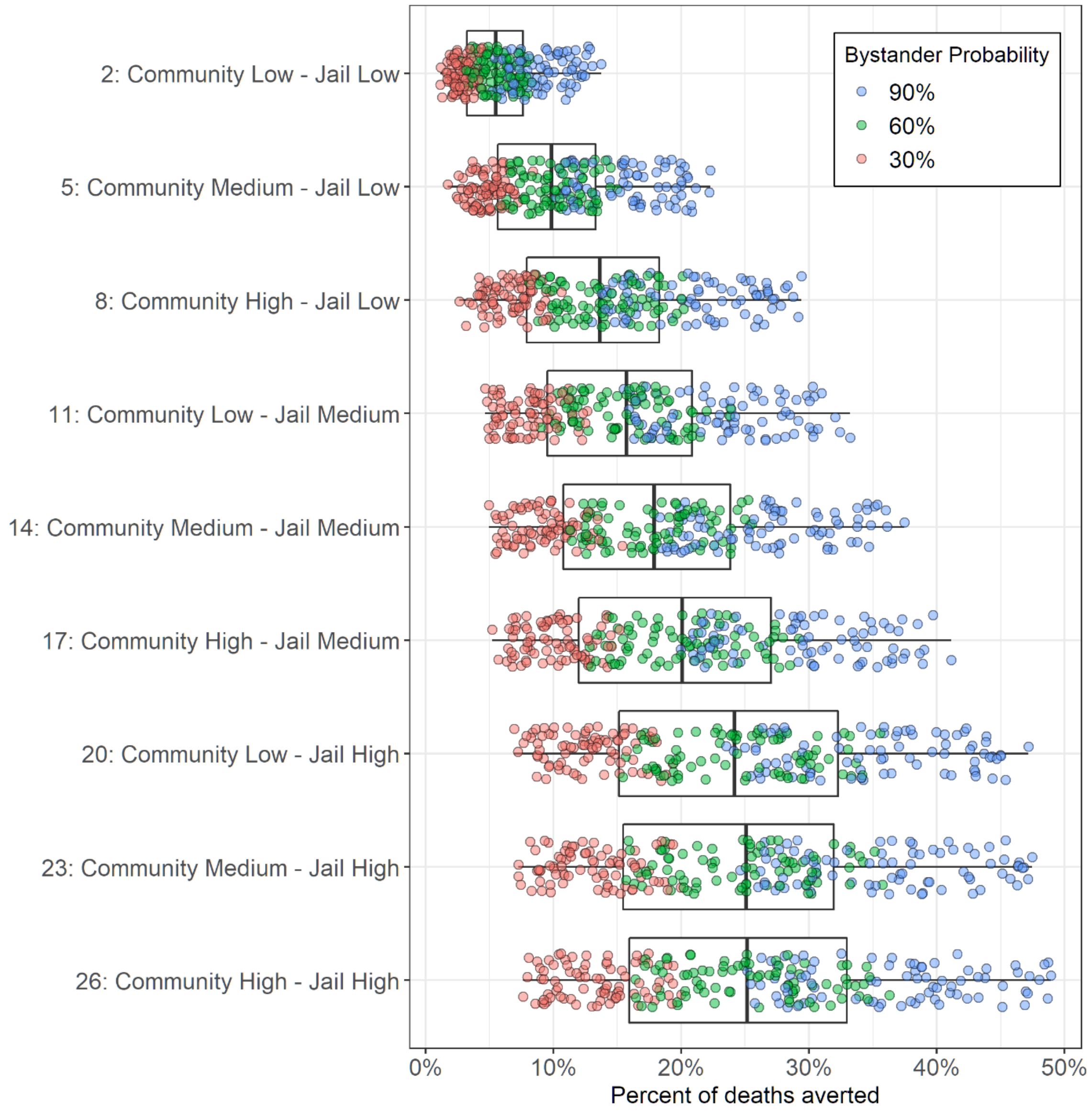

eFigure 1. Deaths Averted for the Jail Release Group for Selected Naloxone Intervention Scenarios That Focus on the Effects of the Community Level and Jail Level Naloxone Distribution

The nine individual boxplots each represent a specific combination of naloxone intervention scenarios, and are labeled by the scenario number and the intervention level (low, medium, high) for the community-based and jail-based intervention strategies. Each colored point represents a unique combination of the non-intervention parameters averaged over ten stochastic runs, colored using the value of bystander probability for the individual run. Box plots represent the median and 25th and 75th percentiles.

© 2024 Tatara E et al. *JAMA Network Open*

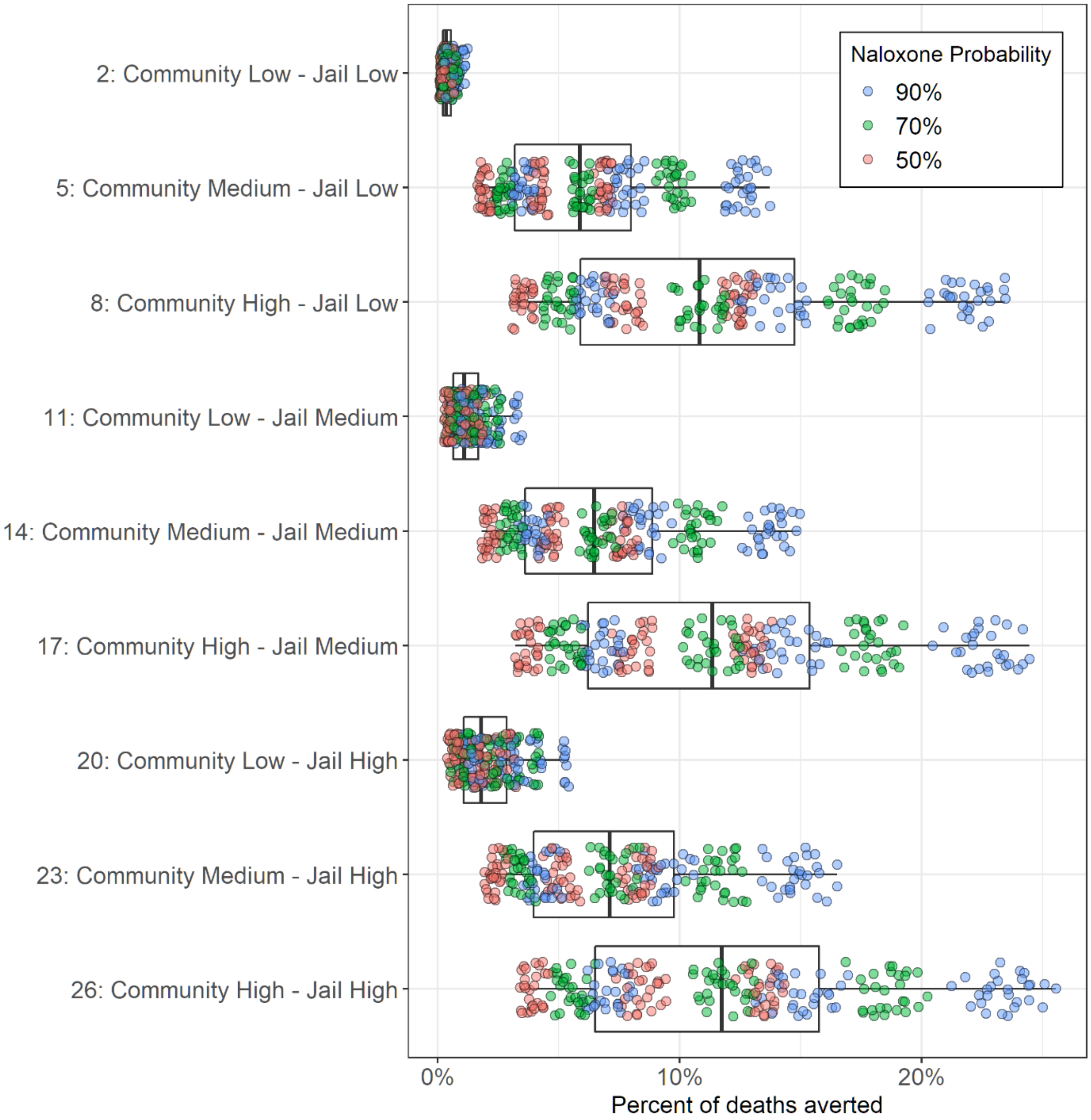

eFigure 2. Percent of Opioid-Related Overdose Deaths Averted for the Entire OU Population for Selected Naloxone Intervention Scenarios

Each circle represents a unique combinations of the non-intervention parameters averaged over ten stochastic runs. Box plots represent the median and 25th and 75th percentiles. Individual simulation points are colored using the value of naloxone administration for the individual run.

© 2024 Tatara E et al. *JAMA Network Open*

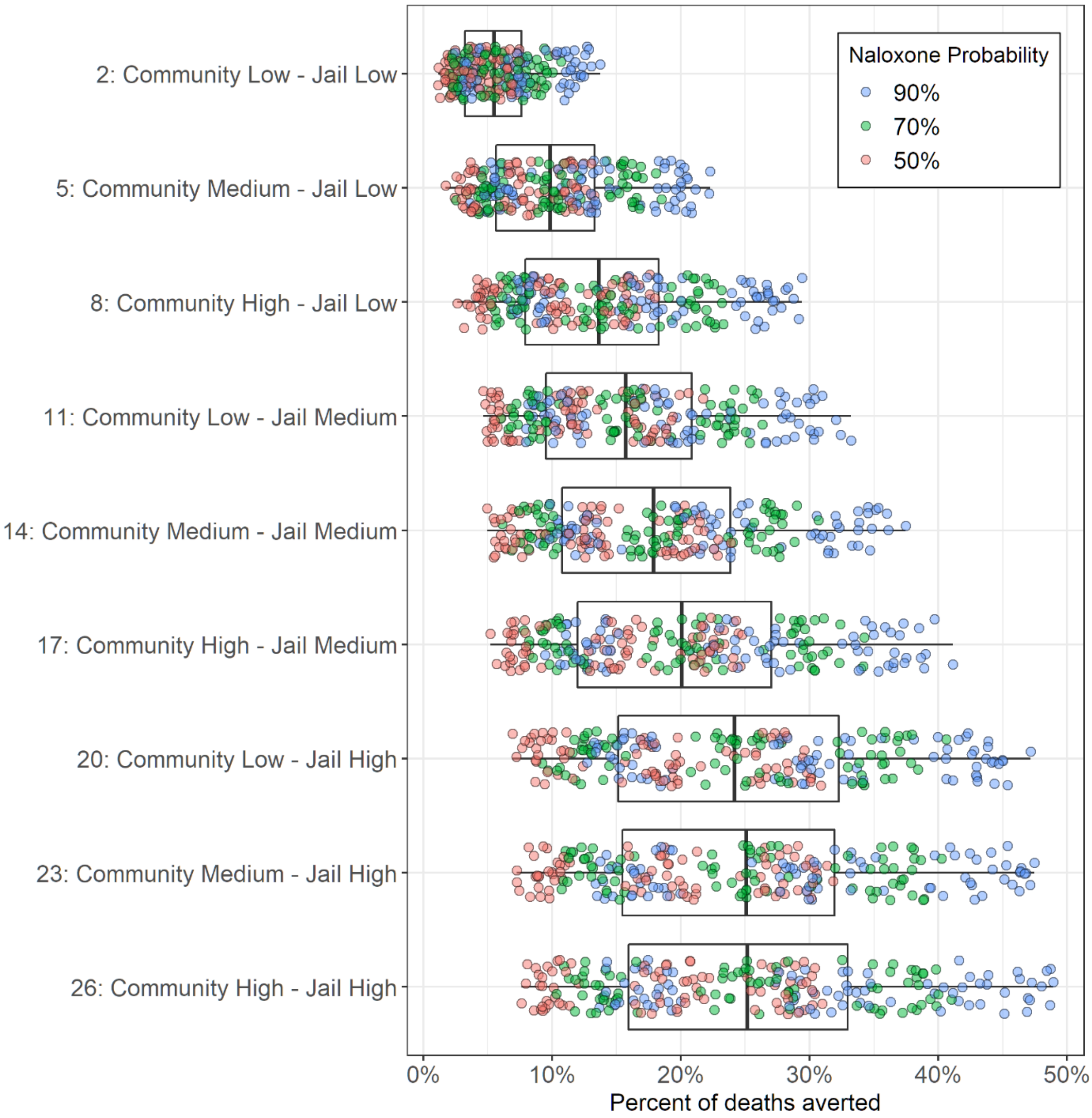

eFigure 3. Percent of Opioid-Related Overdose Deaths Averted for the Jail Release Group (Bottom) for Selected Naloxone Intervention Scenarios

Each circle represents a unique combinations of the non-intervention parameters averaged over ten stochastic runs. Box plots represent the median and 25th and 75th percentiles. Individual simulation points are colored using the value of naloxone administration for the individual run.

© 2024 Tatara E et al. *JAMA Network Open*

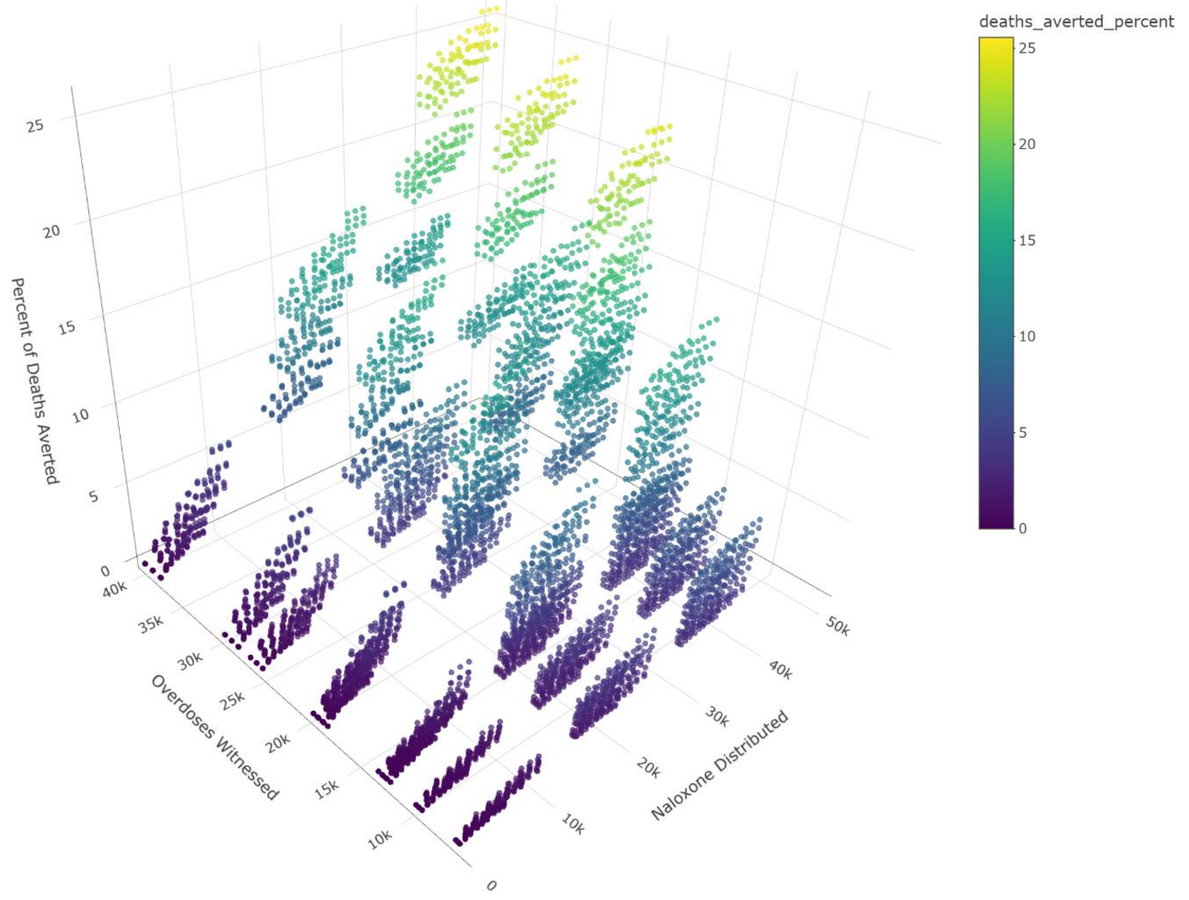

eFigure 4. Scatterplot Showing the Relationship Between the Percent of Deaths Averted in the Entire OU Model Population and the Number of Overdoses Witnessed and the Number of Naloxone Kits Distributed

Each point represents a unique combinations of the non-intervention parameters averaged over ten stochastic runs.

© 2024 Tatara E et al. *JAMA Network Open*

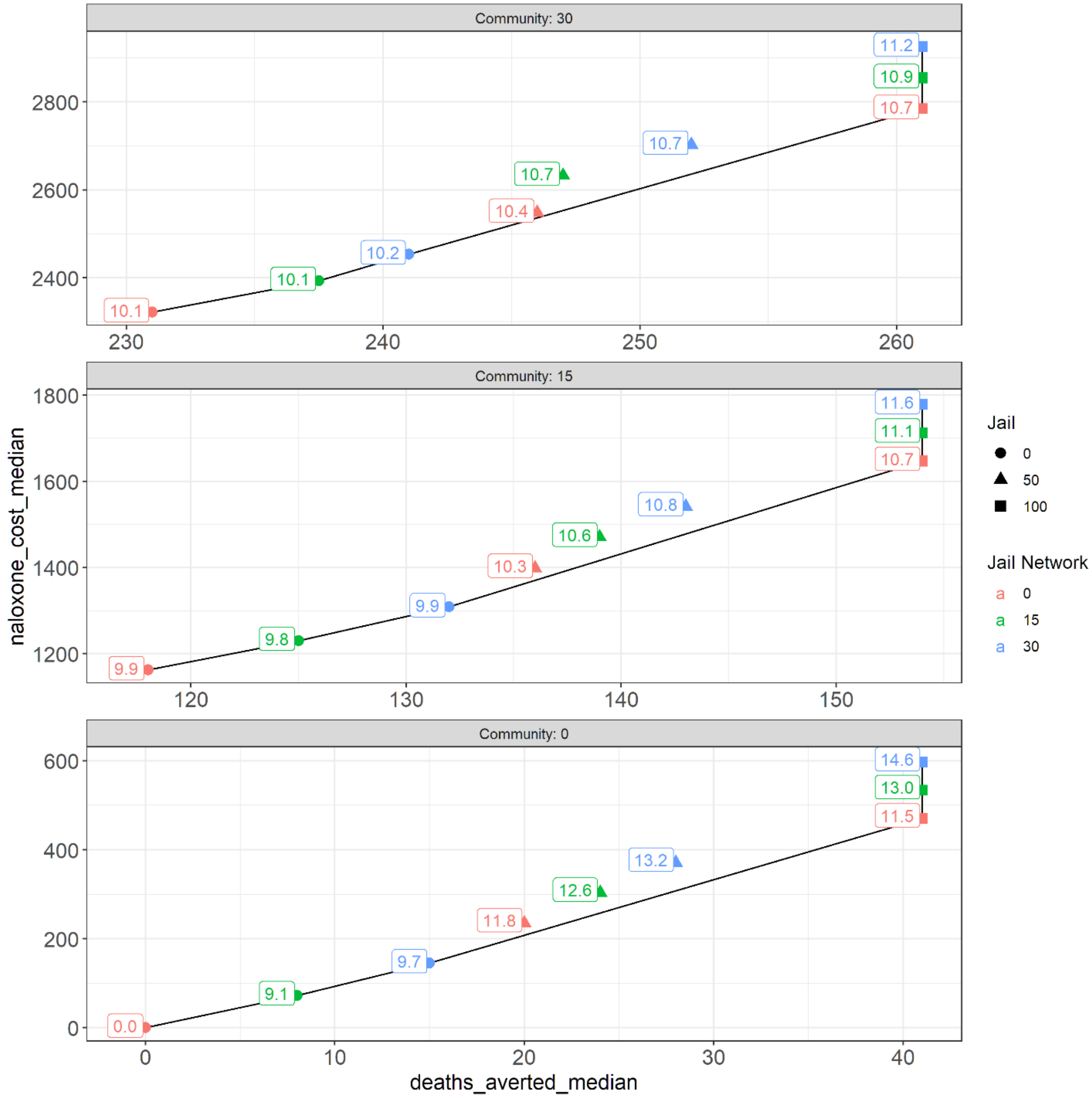

eFigure 5. Median Cost ($1000s) vs Deaths Averted for Each of the 27 Simulated Scenarios as Individual Points, Styled by the Level of Naloxone Distributed via Jail Release Jail and Jail Social Networks (0%, 15%, 30%), and Community Level Naloxone Distribution 0% (bottom), 15% (middle), and 30% (top)

The lines represent the Pareto-optimal front on the convex hull for each of the three community level naloxone distribution levels. Individual points are labeled with the median cost per death averted.

© 2024 Tatara E et al. *JAMA Network Open*

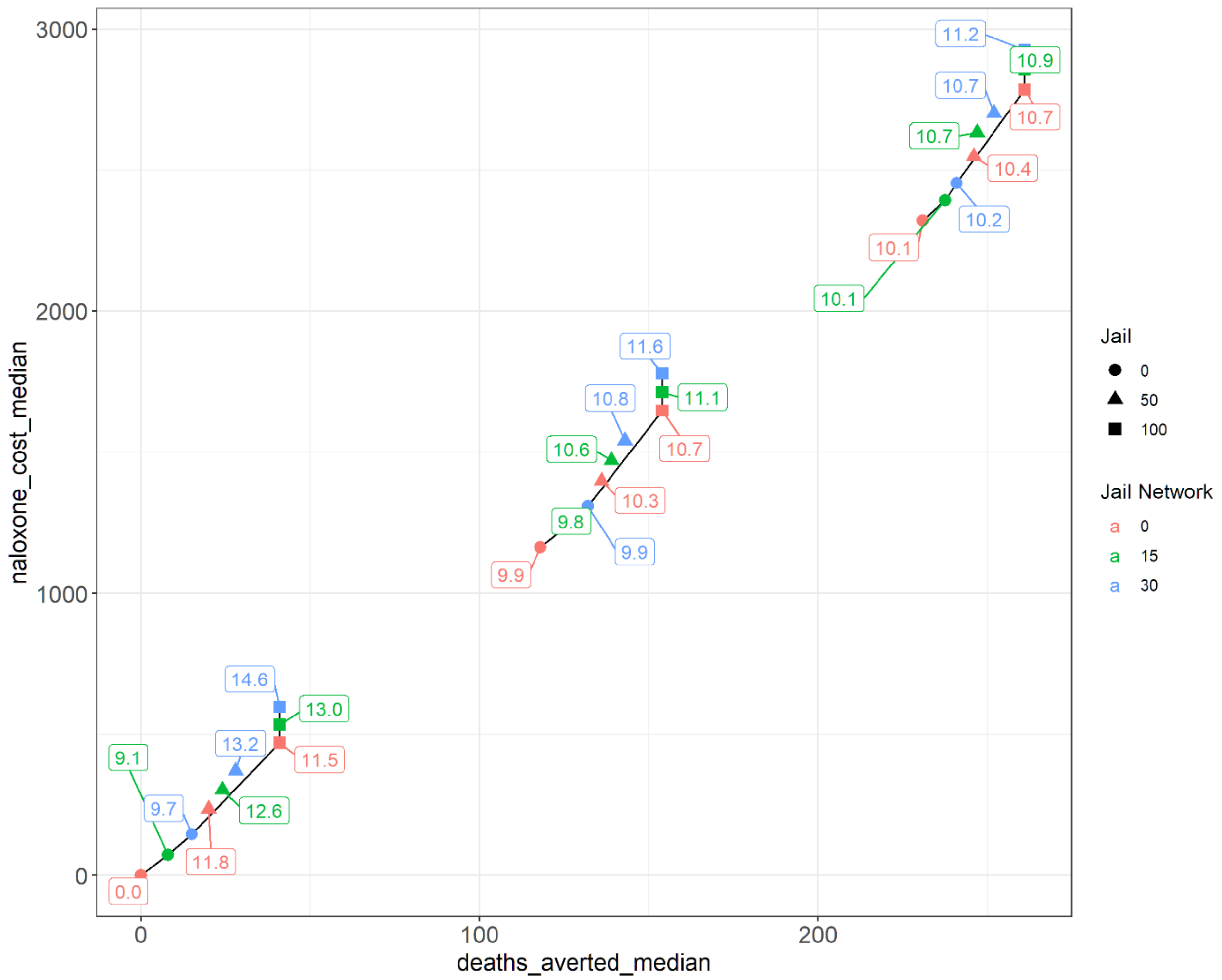

eFigure 6. Median Cost ($1000s) vs Deaths Averted for Each of the 27 Simulated Scenarios as Individual Points, Styled by the Level of Naloxone Distributed via Jail Release Jail and Jail Social Networks (0%, 15%, 30%), and Community Level Naloxone Distribution 0% (left), 15% (middle), and 30% (right)

The lines represent the Pareto-optimal front on the convex hull for each of the three community level naloxone distribution levels. Individual points are labeled with the median cost per death averted.

© 2024 Tatara E et al. *JAMA Network Open*

eReferences

1. Galea S, Riddle M, Kaplan GA. Causal thinking and complex system approaches in epidemiology. International Journal of Epidemiology. 2010 Feb 1;39(1):97–106.

2. Tatara E, Collier NT, Ozik J, Gutfraind A, Cotler SJ, Dahari H, et al. Multi-Objective Model Exploration of Hepatitis C Elimination in an Agent-Based Model of People who Inject Drugs. In: 2019 Winter Simulation Conference (WSC) [Internet]. National Harbor, MD, USA: IEEE; 2019 [cited 2021 Mar 21]. p. 1008–19. Available from: https://ieeexplore.ieee.org/document/9004747/

3. Huo D, Ouellet LJ. Needle exchange and injection-related risk behaviors in Chicago: a longitudinal study. J Acquir Immune Defic Syndr. 2007 May 1;45(1):108–14.

4. Gutfraind A, Boodram B, Prachand N, Hailegiorgis A, Dahari H, Major ME. Agent-Based Model Forecasts Aging of the Population of People Who Inject Drugs in Metropolitan Chicago and Changing Prevalence of Hepatitis C Infections. Kaderali L, editor. PLoS ONE. 2015 Sep 30;10(9):e0137993.

5. Broz D, Ouellet LJ. Prevalence and Correlates of Former Injection Drug Use Among Young Noninjecting Heroin Users in Chicago. Substance Use & Misuse. 2010 Jul 1;45(12):2000–25.

6. Abbasi AB, Salisbury-Afshar E, Jovanov D, Berberet C, Arunkumar P, Aks SE, et al. Health Care Utilization of Opioid Overdose Decedents with No Opioid Analgesic Prescription History. J Urban Health. 2019 Feb;96(1):38–48.

7. Abbasi AB, Salisbury-Afshar E, Berberet CE, Layden JE, Pho MT. Opioid Prescribing Patterns Before Fatal Opioid Overdose. Am J Prev Med. 2020 Feb;58(2):250–3.

8. Chicago Department of Public Health. 2018 Chicago Opioid Overdose Data Brief [Internet]. 2019. Available from: https://www.chicago.gov/content/dam/city/depts/cdph/CDPH/Healthy%20Chicago/ChicagoOpioid2018.pdf

9. The Opioid Crisis in Illinois: Data and the State's Response. Illinois Department of Human Services; 2017.

10. Luo F. State-Level Economic Costs of Opioid Use Disorder and Fatal Opioid Overdose — United States, 2017. MMWR Morb Mortal Wkly Rep [Internet]. 2021 [cited 2021 Oct 28];70. Available from: https://www.cdc.gov/mmwr/volumes/70/wr/mm7015a1.htm

11. Illinois Department of Public Health. Statewide Semiannual Opioid Report [Internet]. 2021 [cited 2021 Oct 28]. Available from: https://dph.illinois.gov/content/dam/soi/en/web/idph/publications/idph/topics-and-services/opioids/idphdata/idph-semiannual-opioid-report-august-2021.pdf

© 2024 Tatara E et al. *JAMA Network Open*

12. Tempalski B, Pouget ER, Cleland CM, Brady JE, Cooper HLF, Hall HI, et al. Trends in the Population Prevalence of People Who Inject Drugs in US Metropolitan Areas 1992–2007. Khudyakov YE, editor. PLoS ONE. 2013 Jun 5;8(6):e64789.

13. Tatara E, Collier NT, Ozik J, Gutfraind A, Cotler SJ, Dahari H, et al. Multi-objective model exploration of hepatitis C elimination in an agent-based model of people who inject drugs. In: Proceedings of the Winter Simulation Conference. National Harbor, Maryland: IEEE Press; 2019. p. 1008–19. (WSC '19).

14. Gutfraind A, Boodram B, Prachand N, Hailegiorgis A, Dahari H, Major ME. Agent-Based Model Forecasts Aging of the Population of People Who Inject Drugs in Metropolitan Chicago and Changing Prevalence of Hepatitis C Infections. PLOS ONE. 2015 Sep 30;10(9):e0137993.

15. Huo D, Ouellet LJ. Needle exchange and injection-related risk behaviors in Chicago: a longitudinal study. J Acquir Immune Defic Syndr. 2007 May 1;45(1):108–14.

16. Tempalski B, Pouget ER, Cleland CM, Brady JE, Cooper HLF, Hall HI, et al. Trends in the Population Prevalence of People Who Inject Drugs in US Metropolitan Areas 1992–2007. Khudyakov YE, editor. PLoS ONE. 2013 Jun 5;8(6):e64789.

17. Lansky A, Abdul-Quader AS, Cribbin M, Hall T, Finlayson TJ, Garfein RS, et al. Developing an HIV Behavioral Surveillance System for Injecting Drug Users: The National HIV Behavioral Surveillance System. Public Health Rep. 2007;122(Suppl 1):48–55.

18. Kelley MS, Chitwood DD. Effects of drug treatment for heroin sniffers: a protective factor against moving to injection? Social Science & Medicine. 2004 May;58(10):2083–92.

19. Ouellet LJ. Patterns and Trends of Drug Abuse in Chicago: 2013. 2014;19.

20. Olson DE, Taheri S. Population Dynamics and the Characteristics of Inmates in the Cook County Jail. Cook County Sheriff's Reentry Council. 2012;9.

21. Cook County Sheriffs [Internet]. [cited 2021 Mar 25]. OpenData Archive > Cook County Sheriffs. Available from: https://www.cookcountysheriff.org/data/

22. Cook County [Internet]. [cited 2021 Oct 28]. Jail Population YOY | Cook County | STAR Performance Management. Available from: https://performance-archive.cookcountyil.gov/Public-Safety/Jail-Population-YOY/t3ix-ahaa

23. Cook County [Internet]. [cited 2021 Oct 28]. Jail Population Exits by Type | Cook County | STAR Performance Management. Available from: https://performance-archive.cookcountyil.gov/Public-Safety/Jail-Population-Exits-by-Type/bdfv-ux2m

© 2024 Tatara E et al. *JAMA Network Open*

24. Eads D. ProPublica. [cited 2021 Mar 25]. How (and Why) We're Collecting Cook County Jail Data. Available from: https://www.propublica.org/nerds/how-and-why-collecting-cook-county-jail-data?token=UvH6WeRiCc8nAyV7r_TRsmoANqWk2uRM

25. WTTW News [Internet]. [cited 2021 Oct 28]. Cook County Jail to Expand Medication-Assisted Treatment for Opioid Addiction. Available from: https://news.wttw.com/2019/05/10/cook-county-jail-expand-medication-assisted-treatment-opioid-addiction

26. Arrestee Drug Abuse Monitoring II (ADAM II) Annual Report. Office of National Drug Control Policy; 2014.

27. Medical Examiner Case Archive | Cook County Open Data [Internet]. [cited 2021 Mar 24]. Available from: https://datacatalog.cookcountyil.gov/Public-Safety/Medical-Examiner-Case-Archive/cjeq-bs86

28. Illinois Department of Public Health. Illinois Opioid Data Dashboard [Internet]. [cited 2021 Mar 24]. Available from: https://idph.illinois.gov/OpioidDataDashboard/

29. Chicago Department of Public Health. Annual Opioid Surveillance Report Chicago 2019 [Internet]. Chicago Department of Public Health; 2021 Mar [cited 2021 Oct 29]. Available from: https://www.chicagohan.org/documents/14171/427549/2019+OPIOID+SURVEILLANCE+REPORT.pdf/3b226c41-6ea3-4136-1376-0b0668a62289?t=1622047952253

30. Chicago Department of Public Health. Chicago Mid-Year Opioid Report 2020 [Internet]. 2020 [cited 2021 Jul 3]. Available from: https://www.chicago.gov/content/dam/city/depts/cdph/tobacco_alchohol_and_drug_abuse/Mid-Year%20Opioid%20Report%202020%20final.pdf

31. Jackson G, Brown A, DeFrances C. Opioid-involved emergency department visits in the National Hospital Care Survey and the National Hospital Ambulatory Medical Care Survey. National Center for Health Statistics; 2020 Dec p. 15. (National Health Statistics Reports). Report No.: 149.

32. Weiss AJ, Barrett ML, Steiner A. Patient Residence Characteristics of Opioid-Related Inpatient Stays and Emergency Department Visits Nationally and by State, 2014. :21.

33. Weiss AJ, Bailey M, O'Malley L, Barrett M, Elixhauser A, Steiner C. Patient Residence Characteristics of Opioid-Related Inpatient Stays and Emergency Department Visits Nationally and by State, 2014 [Internet]. Agency for Healthcare Research and Quality; 2017 Jul p. 21. (Statistical Brief). Report No.: 226. Available from: http://www.hcup-us.ahrq.gov/reports/statbriefs/sb226-Patient-Residence-Opioid-Hospital-Stays-ED-Visits-by-State.pdf

© 2024 Tatara E et al. *JAMA Network Open*

34. Illinois Department of Public Health. Statewide Semiannual Opioid Report [Internet]. 2022 [cited 2022 Oct 10]. Available from: https://dph.illinois.gov/topics-services/opioids/idph-data-dashboard/statewide-semiannual-opioid-report-may-2022.html

35. Chicago Department of Public Health. 2020 Chicago Opioid Overdose Data Brief [Internet]. 2021. Available from: https://www.chicagohan.org/-/alert-details/46669681

36. Cook County Medical Examiner. Cook County Medical Examiner Annual Report [Internet]. 2018. Available from: www.cookcountyil.gov/agency/medical-examiner

37. Cook County Medical Examiner. Cook County Medical Examiner Annual Report [Internet]. 2019 [cited 2021 Oct 29]. Available from: www.cookcountyil.gov/agency/medical-examiner

38. Cook County Medical Examiner. Cook County Medical Examiner Annual Report. 2020;100.

39. State of Illinois Comprehensive Opioid Data Report [Internet]. Illinois Department of Public Health; 2017 [cited 2020 Oct 14]. Available from: https://www.dph.illinois.gov/sites/default/files/publications/publicationsdoil-opioid-data-report.pdf

40. Binswanger I, Blatchford P, Mueller S, Stern M. Mortality after prison release: opioid overdose and other causes of death, risk factors, and time trends from 1999 to 2009. Ann Intern Med. 2013 Nov 5;592–600.

41. Bukten A, Stavseth MR, Skurtveit S, Tverdal A, Strang J, Clausen T. High risk of overdose death following release from prison: variations in mortality during a 15-year observation period: High risk of overdose following release. Addiction. 2017 Aug;112(8):1432–9.

42. Keen C, Young J, Borschmann R, Kinner S. Non-fatal drug overdose after release from prison: A prospective data linkage study. Drug and Alcohol Dependence. 2020 Jan 1;

43. Lim S, Seligson AL, Parvez FM, Luther CW, Mavinkurve MP, Binswanger IA, et al. Risks of Drug-Related Death, Suicide, and Homicide During the Immediate Post-Release Period Among People Released From New York City Jails, 2001-2005. American Journal of Epidemiology. 2012 Mar 15;175(6):519–26.

44. Altekruse SF, Cosgrove CM, Altekruse WC, Jenkins RA, Blanco C. Socioeconomic risk factors for fatal opioid overdoses in the United States: Findings from the Mortality Disparities in American Communities Study (MDAC). Genberg BL, editor. PLoS ONE. 2020 Jan 17;15(1):e0227966.

45. Hickman M. Drug-Related Mortality and Fatal Overdose Risk: Pilot Cohort Study of Heroin Users Recruited From Specialist Drug Treatment Sites in London. Journal of Urban Health: Bulletin of the New York Academy of Medicine. 2003 Jun 1;80(2):274–87.

© 2024 Tatara E et al. *JAMA Network Open*

46. Farrell M, Marsden J. Acute risk of drug-related death among newly released prisoners in England and Wales. Addiction. 2008;103(2):251–5.

47. Pizzicato L, Drake R, Domer-Shank R, Johnson C, Viner K. Beyond the walls: Risk factors for overdose mortality following release from the Philadelphia Department of Prisons. Drug Alcohol Depend. 2018;108–15.

48. Ranapurwala SI, Shanahan ME, Alexandridis AA, Proescholdbell SK, Naumann RB, Edwards D, et al. Opioid Overdose Mortality Among Former North Carolina Inmates: 2000–2015. Am J Public Health. 2018 Sep 1;108(9):1207–13.

49. Sporer K, Kral A. Prescription Naloxone: A Novel Approach to Heroin Overdose Prevention. Annals of Emergency Medicine. 2006 Jul 14;VOLUME 49,(2):172–7.

50. Strang J, McDonald R, Campbell G, Degenhardt L, Nielsen S, Ritter A, et al. Take-Home Naloxone for the Emergency Interim Management of Opioid Overdose: The Public Health Application of an Emergency Medicine. Drugs. 2019;79(13):1395–418.

51. Collier NT, Ozik J, Tatara ER. Experiences in Developing a Distributed Agent-based Modeling Toolkit with Python. In: 2020 IEEE/ACM 9th Workshop on Python for High-Performance and Scientific Computing (PyHPC). 2020. p. 1–12.

52. Collier N, North M. Parallel agent-based simulation with Repast for High Performance Computing. SIMULATION. 2013 Oct 1;89(10):1215–35.

53. Ozik J, Collier NT, Wozniak JM, Spagnuolo C. From desktop to Large-Scale Model Exploration with Swift/T. In: 2016 Winter Simulation Conference (WSC). 2016. p. 206–20.

54. Wozniak JM, Armstrong TG, Wilde M, Katz DS, Lusk E, Foster IT. Swift/T: Large-Scale Application Composition via Distributed-Memory Dataflow Processing. In: 2013 13th IEEE/ACM International Symposium on Cluster, Cloud, and Grid Computing. 2013. p. 95–102.

© 2024 Tatara E et al. *JAMA Network Open*
