# Supplementary material for: Agent-Based Model of Combined Community- and Jail-Based Take-Home Naloxone Distribution
Source: JAMA Netw Open. 2024 Dec 10;7(12):e2448732. doi: 10.1001/jamanetworkopen.2024.48732 (PMC11632540; doi:10.1001/jamanetworkopen.2024.48732)
Supplement: Supplement 2. — Data Sharing Statement [file jamanetwopen-e2448732-s002.pdf]

Data Sharing Statement

Tatara. Agent-Based Model of Combined Community- and Jail-Based Take-Home Naloxone Distribution. *JAMA Netw Open*. Published December 10, 2024.
doi:10.1001/jamanetworkopen.2024.48732

Data
Data available: Yes
Data types: Data (not involving human participants), Other (please specify)
Additional Information: We will provide all parameters used to calibrate our simulation models.
How to access data: tatara@anl.gov
When available: With publication

Supporting Documents
Document types: None

Additional Information
Who can access the data: Anyone requesting the data
Types of analyses: for any purpose
Mechanisms of data availability: with investigator support
Any additional restrictions: None
